# Supplementary material for: Broad geographical circulation of a novel vesiculovirus in bats in the Mediterranean region
Source: PLoS Negl Trop Dis. 2025 Jun 12;19(6):e0013172. doi: 10.1371/journal.pntd.0013172 (PMC12193708; doi:10.1371/journal.pntd.0013172)
Supplement: S1 Fig — The first PCR round is based on the primers F1_Rhabdovirus (forward, 5’-ATWGGNYTNAARSSIAARGA-3’) and R1_Rhabdovirus (reverse, 5’-RYYTGRTTRTCNCCYTGIGC-3’), whereas the nested PCR round is based on primer F2_Rhabdovirus-M (forward, 5’-GAYTAYGANAARTGGAAYAAYYAYCA-3’) and R2_Rhabdovirus (reverse, 5’-TGYCKNARNCCYTCYARNCCICC-3’). The nucleotide code applied is: R = A/G, Y = C/T, M = A/C, K = G/T, S = G/C, W = A/T, H = A/T/C, B = G/T/C, V = G/A/C, D = G/A/T, N = A/T/C/G and I (Hypoxanthine). The oligonucleotide sequence of each primer is indicated in bold, together with its name and an arrow indicating the sense direction. Sequence identity of the primers based on the multiple alignment is highlighted in black. The dot lines between the different blocks of sequence represent omitted regions. The position of the primers is indicated according to the nucleotide sequence of the polymerase gene of Drosophila obscura sigmavirus (DObSV) (GenBank accession number NC_022580). Virus acronyms and genera are indicated in the left of the figure. The multiple alignment was performed with ClustalW, version 2.0. (DOCX) [file pntd.0013172.s001.docx]

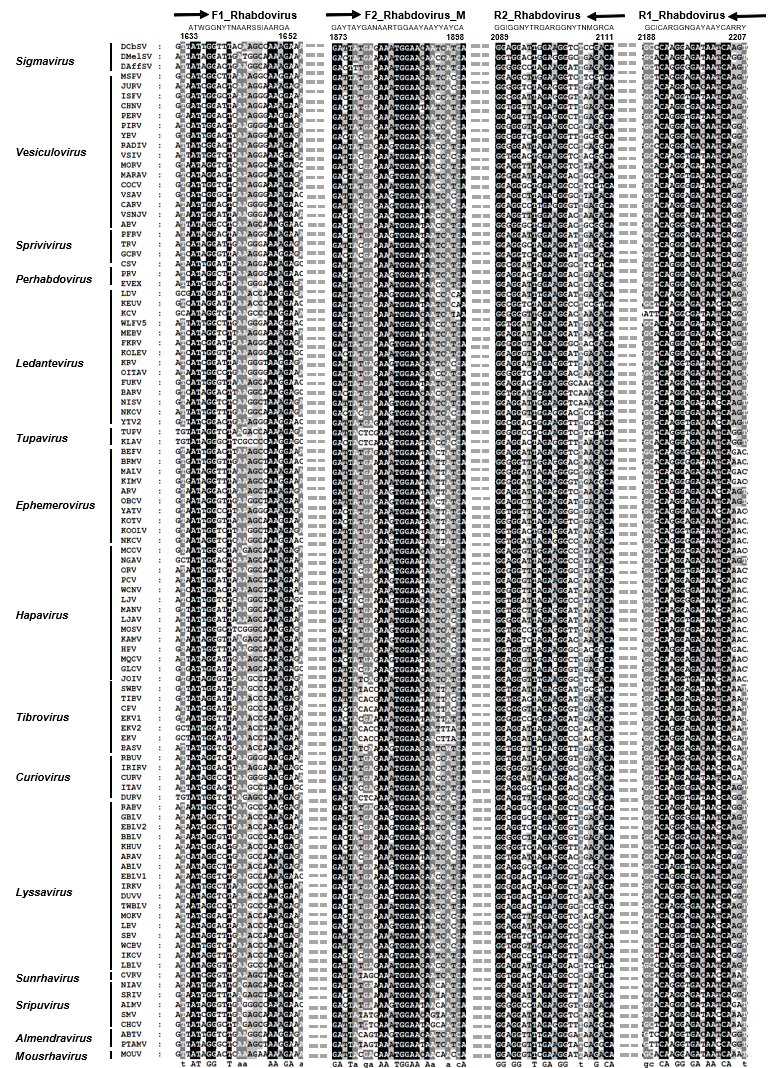
**Supplementary Figure 1.** Multiple alignment of 103 nucleotide coding sequences of the polymerase sequences of rhabdoviruses belonging to 14 different genera within the family *Rhabdoviridae*, with the position and the sequences of the four primers F1_Rhabdovirus, R1_Rhabdovirus, F2_Rhabdovirus-M and R2_Rhabdovirus selected for the pan-rhabdo RT-nqPCR.

The first PCR round is based on the primers F1_Rhabdovirus (forward, 5’- ATWGGNYTNAARSSIAARGA-3’) and R1_Rhabdovirus (reverse, 5’-RYYTGRTTRTCNCCYTGIGC-3’), whereas the nested PCR round is based on primer F2_Rhabdovirus-M (forward, 5’-GAYTAYGANAARTGGAAYAAYYAYCA-3’) and R2_Rhabdovirus (reverse, 5’-TGYCKNARNCCYTCYARNCCICC-3’). The nucleotide code applied is: R=A/G, Y=C/T, M=A/C, K=G/T, S=G/C, W=A/T, H=A/T/C, B=G/T/C, V=G/A/C, D=G/A/T, N=A/T/C/G and I (Hypoxanthine). The oligonucleotide sequence of each primer is indicated in bold, together with its name and an arrow indicating the sense direction. Sequence identity of the primers based on the multiple alignment is highlighted in black. The dot lines between the different blocks of sequence represent regions which were omitted in the figure. The position of the primers is indicated according to the nucleotide sequence of polymerase gene of Drosophila obscura sigmavirus (DObSV) (GenBank accession number NC_022580). Virus acronyms and genera are indicated in the left of the figure. The multiple alignment was performed with ClustalW, version 2.0.
