# Supplementary material for: Broad geographical circulation of a novel vesiculovirus in bats in the Mediterranean region
Source: PLoS Negl Trop Dis. 2025 Jun 12;19(6):e0013172. doi: 10.1371/journal.pntd.0013172 (PMC12193708; doi:10.1371/journal.pntd.0013172)
Supplement: S2 Fig — After RNA extraction of the samples, a first cDNA step is performed, followed by a 1st conventional PCR. A nested qPCR is systematically performed, and results are interpreted according to positive and negative samples, based on the shape of the melting curve and the value of the Tm. Positive samples are confirmed with an additional 2nd round of conventional PCR (from the 1st round conventional PCR). Amplicons are analyzed after migration on gel electrophoresis, and positive samples are Sanger sequenced. Validation of positive results is obtained after BLASTn and/or BLASTx analysis. Alternatively, amplicons from 2nd round of qPCR can also be directly Sanger sequenced and analyzed by BLAST. Templates (cDNA) from tissue samples need to be diluted 1:10 in nuclease-free water before the 1st round of conventional PCR. This dilution step is not required for liquid samples (e.g. blood or oral swab). (DOCX) [file pntd.0013172.s002.docx]

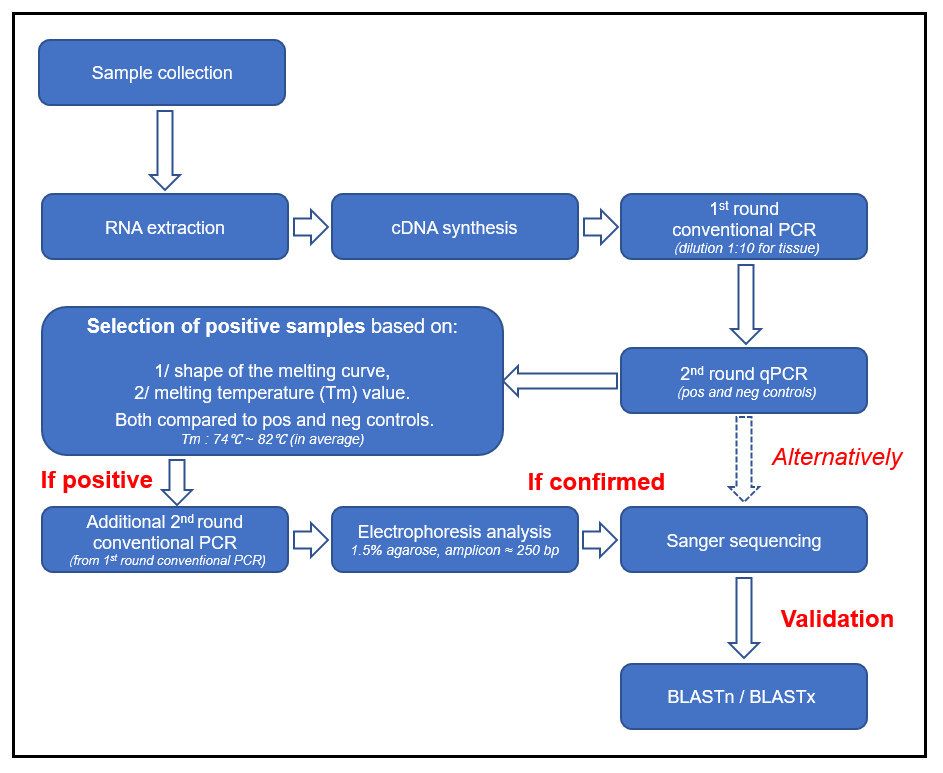


**Supplementary Figure 2.** Workflow analysis for the interpretation of the results obtained with the pan-rhabdo RT-nqPCR. After RNA extraction of the samples, the first cDNA step is performed, followed by a 1^st^ conventional PCR. A nested qPCR is systematically performed, and results are interpreted according to positive and negative samples, based on the shape of the melting curve and the value of the Tm. Positive samples are confirmed with an additional 2^nd^ round conventional PCR (from the 1^st^ round conventional PCR). Amplicons are analyzed after gel electrophoresis migration, and positive samples are Sanger sequenced. Validation of positive results is obtained after BLASTn and/or BLASTx analysis. Alternatively, amplicons from the 2^nd^ round qPCR can also be directly Sanger sequenced and analyzed by BLAST. Templates (cDNA) from tissue samples request a 1:10 dilution in nuclease-free water before proceeding to the 1^st^ round of conventional PCR. This dilution step is not required for liquid samples (e.g. blood or oral swab).
