# Supplementary material for: Broad geographical circulation of a novel vesiculovirus in bats in the Mediterranean region
Source: PLoS Negl Trop Dis. 2025 Jun 12;19(6):e0013172. doi: 10.1371/journal.pntd.0013172 (PMC12193708; doi:10.1371/journal.pntd.0013172)
Supplement: S3 Fig — Dot lines indicate the conserved regions not shown in the figure. A total of 28 amino acid mutations were identified and represented. The multiple alignment was performed with ClustalW 2.0. (DOCX) [file pntd.0013172.s003.docx]

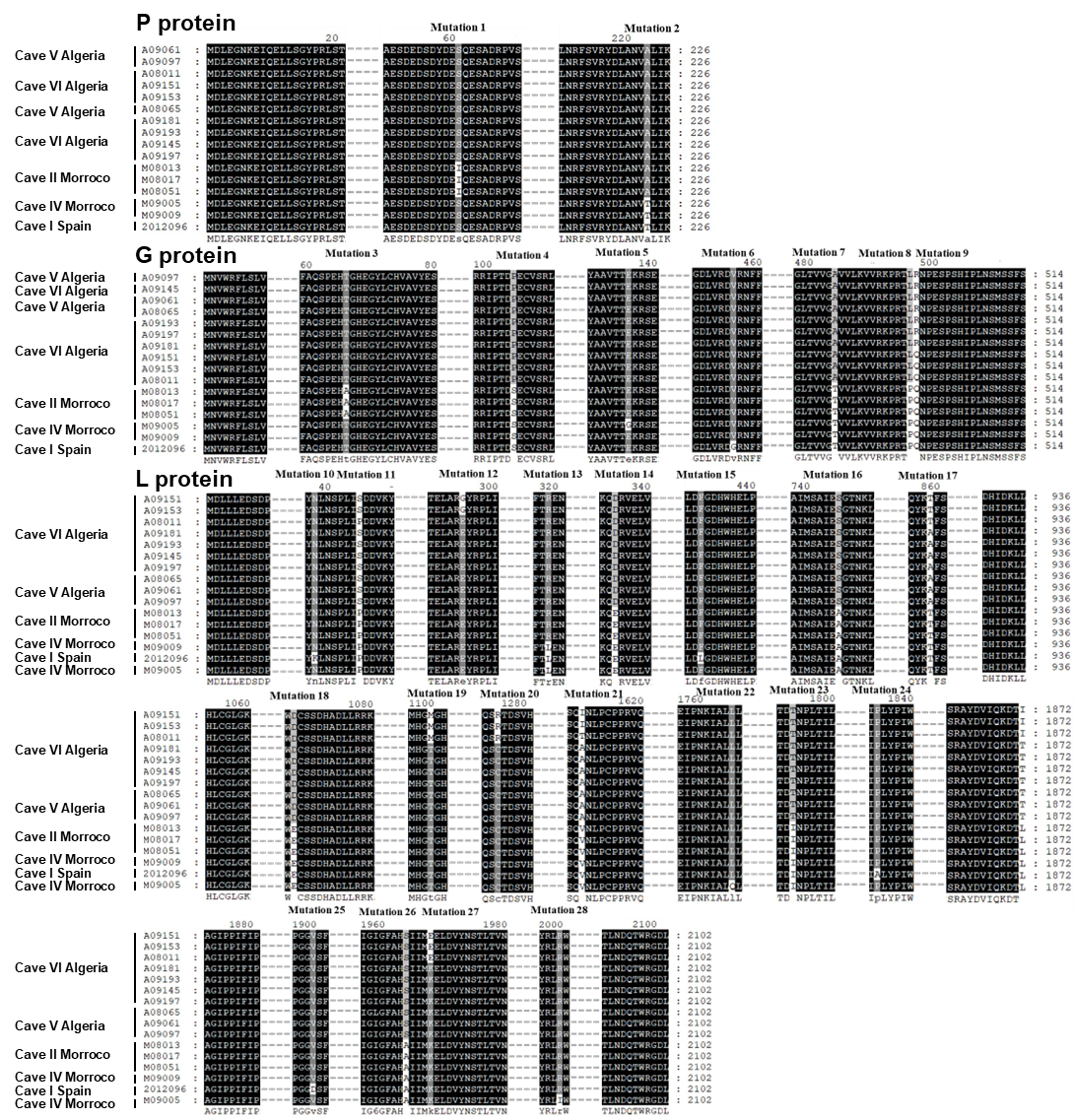


**Supplementary Figure 3.** Identification of the different deducted amino acid mutations between the 16 isolates of Mediterranean bat virus (MBV), according to the geographical location of the caves. Dot lines indicate the conserved regions not shown in the figure. A total of 28 amino acid mutations were identified and represented. The multiple alignment was performed with ClustalW 2.0.
