# Supplementary material for: Broad geographical circulation of a novel vesiculovirus in bats in the Mediterranean region
Source: PLoS Negl Trop Dis. 2025 Jun 12;19(6):e0013172. doi: 10.1371/journal.pntd.0013172 (PMC12193708; doi:10.1371/journal.pntd.0013172)
Supplement: S1 Text — (DOCX) [file pntd.0013172.s004.docx]

S1_Text : Supplementary text

Broad geographical circulation of a novel vesiculovirus in bats in the Mediterranean region

Dong-Sheng Luo^1,2*^, Markéta Harazim^3^, Corinne Maufrais^4^, Simon Bonas^1^, Natalia Martinkova^3^, Aude Lalis^5^, Emmanuel Nakouné^6^, Edgard Valéry Adjogoua^7^, Mory Douno^8^, Blaise Kadjo^9^, Marc López-Roig^10,11^, Jiri Pikula^12^, Zheng-Li Shi^13^, Hervé Bourhy^1^, Jordi Serra-Cobo^10,11^ and Laurent Dacheux^1¶*^

^1^Institut Pasteur, Université Paris Cité, Unit Lyssavirus Epidemiology and Neuropathology, Paris, France

^2^Department of Infection Biology, London School of Hygiene & Tropical Medicine, WC1E 7HT, London, UK

^3^Institute of Vertebrate Biology, The Czech Academy of Sciences, Brno, Czechia

^4^Institut Pasteur, Université Paris Cité, Bioinformatics and Biostatistics Hub, Paris, France

^5^Institut de Systématique, Evolution, Biodiversité (ISYEB), Muséum National d’Histoire Naturelle, CNRS, SU, EPHE-PSL, UA, Paris, France

^6^Virology Department, Institut Pasteur of Bangui, Bangui, Central African Republic

^7^Department of Epidemic Viruses, Institut Pasteur of Cote d'Ivoire, Abidjan, Cote d'Ivoire

^8^Centre de Gestion de l'Environnement des monts Nimba et Simandou (CEGENS) Lola / Ministère de l'Environnement et du Développement Durable, Conakry, Guinée-Conakry

^9^Université Félix-Houphouët-Boigny, UFR Biosciences, Abidjan, Cote d'Ivoire

^10^ Departament de Biologia Evolutiva, Ecologia i Ciències Ambientals, Facultat de Biologia, Barcelona, Spain

^11^Institut de Reserca de Biodiversitat (IRBio), Universitat de Barcelona, Barcelona, Spain

^12^Department of Ecology and Diseases of Zoo Animals, Game, Fish and Bees, University of Veterinary Sciences Brno, Brno, Czechia

^13^Guangzhou National Laboratory, No. 9 XingDaoHuanBei Road, Guangzhou International Bio Island, Guangzhou, China.

^¶^ Present address: Institut Pasteur, Université Paris Cité, Unit Environnement and Infectious Risks, Paris, France

*** Correspondence:**

Dongsheng Luo

[DongshengLuo@outlook.com](mailto:DongshengLuo@outlook.com)

Laurent Dacheux

[laurent.dacheux@pasteur.fr](mailto:laurent.dacheux@pasteur.fr)

# Supplementary Materials and Methods

## Pan-rhabdovirus PCR method validation

Primers were tested by both conventional PCR and SYBR Green-based real-time PCR (qPCR). Conventional PCR was performed with the TaKaRa EX Taq kit (TaKaRa) and included 5 µL 10X PCR Buffer (Mg^2+^ plus), 4.0 µL dNTP Mix (2.5 mM each), 2.0 µL of both forward and reverse primers (200 µM), 2 µL of complementary DNA (cDNA) template and 5 µL of nuclease free water under a final volume of 50 µL. SYBR Green-based real-time PCR (qPCR) was carried out with the Power SYBR Green Master Mix kit (ThermoFisher) using 12.5 µL Mix Reagent, 1 µL forward and reverse primers (200 µM), 2 µL cDNA template and completed to 25 µL with nuclease free water. Thermocycling conditions were performed according to the manufacturer’s instructions, and the annealing temperature (Tm) for each PCR system was calculated by TM calculator (http//www.Thermofisher.com).

## Virus genome sequencing

Host ribosomal RNA was removed to 2-4 µg of total RNA with 1 µL of Terminator 5’-Phosphate-Dependent Exonuclease (Epicentre Biotechnologies), in addition to 2 µL of buffer A and 0.5 µL of RNAsin Ribonuclease inhibitor (Promega). After adjusting to 20 µL with nuclease free water, the mix was incubated for 1 h at 30°C. After purification using Agencourt RNAclean XP beads (Beckman Coulter) at ratio 1:1.8, following the manufacturer’s instructions, cDNA synthesis was performed using 8 µL of RNA and the Superscript III reverse transcriptase (Invitrogen) according to manufacturer’s instructions. The first incubation was done at 70°C for 5 min after adding 1 μL of 10 mM dNTP mix (Invitrogen) and 1 μL of 50 μM of random hexamers (Invitrogen). Samples were then placed on ice, and the complementary step was done with the addition of 1 μL (200 U) of Superscript III reverse transcriptase (Invitrogen), 2 μL of 10x First-Strand Reaction Buffer, 2 μL of 0.1 DTT, 4 μL of 25 mM MgCl_2_, and 1 μL of RNAsin Ribonuclease inhibitor (Promega) for a final volume of 20 μL. The mix was incubated at 25°C for 10 min then at 50°C for 90 min. Afterward, double-stranded DNA (dsDNA) synthesis was performed by adding to the 20 μL fresh cDNA a mix reaction containing 10x Second-Strand Synthesis Reaction Buffer (New England Biolabs), 3 μL of 10 mM dNTP mix (Invitrogen), 1 μL (10 U) of *E. coli* DNA ligase, 4 μL (40 U) of *E. coli* DNA polymerase I, 1 µL (5 U) of *E. coli* RNase H (New England Biolabs) and 43 µL of nuclease-free water. After incubation at 16°C for 2 h, the total volume (80 µL) of dsDNA was purified using a ratio of 1:1.8 of AMPure XP beads (Beckman Coulter) following the manufacturer’s instructions. dsDNA libraries were constructed for using the Nextera XT kit (Illumina) and sequenced using a 2 × 150 nucleotide paired-end strategy on the NextSeq500 platform housed in Institut Pasteur.
