# Supplementary material for: Broad geographical circulation of a novel vesiculovirus in bats in the Mediterranean region
Source: PLoS Negl Trop Dis. 2025 Jun 12;19(6):e0013172. doi: 10.1371/journal.pntd.0013172 (PMC12193708; doi:10.1371/journal.pntd.0013172)
Supplement: S1 Table — (DOCX) [file pntd.0013172.s005.docx]

**Table S1.** Details of the bat samples included in this study, according to the species and to the type of samples (brain, oral swab and blood).

| **Bat** | |  | **Sample type (No.)** | | | **Total (No.)** |
| --- | --- | --- | --- | --- | --- | --- |
| **Family** | **Species** |  | **Brain** | **Oral swab** | **Blood** |  |
| Pteropodidae | *Rousettus aegyptiacus* |  |  |  | 100 | 100 |
|  | *Eidolon helvum* |  | 222 |  |  | 222 |
|  | *Epomophorus gambianus* |  | 5 | 7 |  | 12 |
|  | *Epomops buettikoferi* |  | 3 | 2 |  | 5 |
|  | *Epomops franqueti* |  | 3 |  |  | 3 |
|  | *Epomops sp.* |  | 1 | 4 |  | ~~5~~ |
|  | *Hypsignathus gambianus* |  | 1 |  |  | 1 |
|  | *Hypsignathus monstrosus* |  | 5 |  |  | 5 |
|  | *Megaloglossus azagnyi* |  | 1 |  |  | 1 |
|  | *Micropteropus pusilillus* |  | 99 | 41 |  | 140 |
|  | *Myonycteris leptodon* |  | 4 | 3 |  | 7 |
|  | *Myonycteris torquata* |  | 1 | 3 |  | 4 |
|  | *Nanonycteris veldkampii* |  | 4 | 1 |  | 5 |
|  | *Scotonycteris zenkeri* |  | 4 |  |  | 4 |
| Rhinolophidae | *Rhinolophus alcyone* |  | 3 | 2 |  | 5 |
|  | *Rhinolophus blasii* |  |  |  | 1 | 1 |
|  | *Rhinolophus euryale* |  |  |  | 41 | 41 |
|  | *Rhinolophus ferrumequinum* |  | 11 |  | 115 | 126 |
|  | *Rhinolophus hipposideros* |  |  |  | 1 | 1 |
|  | *Rhinolophus sp.* |  | 2 | 1 | 6 | 9 |
| Hipposideridae | *Hipposideros caffer* |  | 27 | 6 |  | 33 |
|  | *Hipposideros ruber* |  | 3 | 1 |  | 4 |
|  | *Hipposideros sp.* |  | 2 | 1 |  | 3 |
| Rhinopomatidae | *Rhinopoma hardwickii* |  |  |  | 7 | 7 |
|  | *Rhinopoma microphyllum* |  |  |  | 21 | 21 |
| Miniopteridae | *Miniopterus schreibersii* |  | 4 | 193 | 157 | 354 |
| Vespertilionidae | *Myotis blythii* |  |  | 1 | 12 | 13 |
|  | *Myotis capaccinii* |  |  | 7 | 7 | 14 |
|  | *Myotis dasycneme* |  | 1 |  |  | 1 |
|  | *Myotis emarginatus* |  | 3 |  | 27 | 30 |
|  | *Myotis escalerai* |  |  | 9 | 1 | 10 |
|  | *Myotis myotis* |  | 188 | 131 |  | 319 |
|  | *Myotis mystacinus* |  | 12 |  |  | 12 |
|  | *Myotis nattereri* |  | 1 |  |  | 1 |
|  | *Myotis punicus* |  | 1 |  | 297 | 298 |
|  | *Afronycteris nana* |  | 2 | 2 |  | 4 |
|  | *Noctulas noctula* |  | 48 |  |  | 48 |
|  | *Nyctalus leisleri* |  | 1 |  |  | 1 |
|  | *Nycteris grandis* |  | 1 | 1 |  | 2 |
|  | *Nycteris hispida* |  | 1 | 1 |  | 2 |
|  | *Nycteris thebaica* |  | 2 | 2 |  | 4 |
|  | *Nycteris sp.* |  | 2 |  |  | 2 |
|  | *Pipistrellus kuhlii* |  | 5 |  | 2 | 7 |
|  | *Pipistrellus pipistrellus* |  | 7 |  |  | 7 |
|  | *Scotophilus leucogaster* |  | 4 | 4 |  | 8 |
|  | *Vespertilio murinus* |  | 5 |  |  | 5 |
|  | *Eptesicus isabellinus* |  |  |  | 3 | 3 |
|  | *Eptesicus serotinus* |  | 32 |  |  | 32 |
| Emballonuridae | *Taphozous nudiventris* |  |  |  | 18 | 18 |
| Molossidae | *Molossus molossus* |  | 2 |  |  | 2 |
| **Total** | |  | 723 | 423 | 816 | 1962 |
