# Supplementary material for: Broad geographical circulation of a novel vesiculovirus in bats in the Mediterranean region
Source: PLoS Negl Trop Dis. 2025 Jun 12;19(6):e0013172. doi: 10.1371/journal.pntd.0013172 (PMC12193708; doi:10.1371/journal.pntd.0013172)
Supplement: S2 Table — (DOCX) [file pntd.0013172.s006.docx]

**Table S2**. Description of the animal rhabdoviruses selected to design the primers of the pan-rhabdo RT-nqPCR based on the polymerase gene sequence.

| **Virus** | **Species** | **Genus** | **Location of first isolation** | **Year of isolation** | **Source species** | **GenBank accession** |
| --- | --- | --- | --- | --- | --- | --- |
| Arboretum virus (ABTV) | *Almendravirus arboretum* | \| *Almendravirus* \| \| --- \| | Peru | \| 2009 \| \| --- \| | mosquitoe | NC_025393 |
| Puerto Almendras virus (PTAMV) | *Almendravirus almendras* | *Almendravirus* | Peru | 2009 | mosquitoe | NC_025395 |
| Curionopolis virus (CURV) | *Curiovirus curionopolis* | \| *Curiovirus* \| \| --- \| | Brazil | \| 1985 \| \| --- \| | midge | NC_025354 |
| Iriri virus (IRIRV) | *Curiovirus iriri* | \| *Curiovirus* \| \| --- \| | Brasil | \| 1982 \| \| --- \| | sandfly | NC_034544 |
| Itacaiunas virus (ITAV) | *Curiovirus itacaiunas* | \| *Curiovirus* \| \| --- \| | Brazil | \| 1984 \| \| --- \| | midge | NC_034536 |
| Rochambeau virus (RBUV) | *Curiovirus rochambeau* | \| *Curiovirus* \| \| --- \| | French Guiana | 1973 | \| mosquitoe \| \| --- \| | \| NC_034534 \| \| --- \| |
| \| 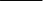Adelaide River virus (ARV) \| \| --- \| | \| *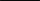Ephemerovirus adelaide* \| \| --- \| | \| *Ephemerovirus* \| \| --- \| | Australia | \| 1981 \| \| --- \| | bovine | NC_028246 |
| Berrimah virus (BRMV) | *Ephemerovirus berrimah* | *Ephemerovirus* | Australia | 1981 | bovine | NC_025358 |
| Bovine ephemeral fever virus (BEFV) | *Ephemerovirus febris* | *Ephemerovirus* | China | 2002 | bovine | KY315724 |
| Kimberley virus (KIMV) | *Ephemerovirus kimberley* | *Ephemerovirus* | Australia | 1980 | bovine | NC_025396 |
| Koolpinyah virus (KOOLV) | *Ephemerovirus koolpinyah* | \| *Ephemerovirus* \| \| --- \| | Australia | \| 1985 \| \| --- \| | bovine | NC_028239 |
| Kotonkan virus (KOTV) | *Ephemerovirus kotonkan* | *Ephemerovirus* | Nigeria | 1967 | midge | NC_017714 |
| Malakal virus (MALV) | *Ephemerovirus kimberley* | *Ephemerovirus* | Sudan | 1963 | mosquitoe | JQ941707 |
| New Kent County virus (NKCV) | *Ephemerovirus kent* | *Ephemerovirus* | USA | 2016 | tick | MF615270 |
| Obodhiang virus (OBOV) | *Ephemerovirus obodhiang* | *Ephemerovirus* | Sudan | 1963 | mosquitoe | NC_017685 |
| Yata virus (YATV) | *Ephemerovirus yata* | \| *Ephemerovirus* \| \| --- \| | Central African Republic | \| 1969 \| \| --- \| | mosquitoe | NC_028241 |
| Manitoba virus (MANV) | *Hapavirus manitoba* | \| *Hapavirus* \| \| --- \| | Canada | \| 1977 \| \| --- \| | mosquitoe | NC_034531 |
| Ngaingan virus (NGAV) | *Hapavirus ngaingan* | \| *Hapavirus* \| \| --- \| | Australia | \| 1970 \| \| --- \| | midge | NC_013955 |
| Gray Lodge virus (GLOV) | *Hapavirus graylodge* | \| *Hapavirus* \| \| --- \| | USA | \| 1971 \| \| --- \| | mosquitoe | NC_034541 |
| Hart Park virus (HPV) | *Hapavirus hartpark* | *Hapavirus* | USA | 1955 | mosquitoe | NC_034447 |
| Joinjakaka virus (JOIV) | *Hapavirus joinjakaka* | *Hapavirus* | Papua-New Guinea | 1966 | mosquitoes | NC_034538 |
| Kamese virus (KAMV) | *Hapavirus kamese* | *Hapavirus* | Central African Republic | 1977 | mosquitoe | KX497133 |
| La Joya virus (LJV) | *Hapavirus lajoya* | \| *Hapavirus* \| \| --- \| | Panama | \| 1958 \| \| --- \| | mosquitoe | NC_034537 |
| Landjia virus (LJAV) | *Hapavirus landjia* | \| *Hapavirus* \| \| --- \| | Central African Republic | \| 1970 \| \| --- \| | bird | NC_034533 |
| Marco virus (MCOV) | *Hapavirus marco* | \| *Hapavirus* \| \| --- \| | Brazil | \| 1962 \| \| --- \| | lizard | NC_034530 |
| Mosqueiro virus (MQOV) | *Hapavirus mosqueiro* | *Hapavirus* | Brazil | 1970 | mosquitoe | NC_034448 |
| Mossuril virus (MOSV) | *Hapavirus mossuril* | \| *Hapavirus* \| \| --- \| | Mozambique | \| 1959 \| \| --- \| | mosquitoe | KM204993 |
| Ord River virus (ORV) | *Hapavirus ord* | *Hapavirus* | Australia | 1976 | mosquitoe | KY421920 |
| Parry Creek virus (PCV) | *Hapavirus parry* | *Hapavirus* | Australia | 1973 | mosquitoe | NC_034449 |
| Wongabel virus (WONV) | *Hapavirus wongabel* | *Hapavirus* | Australia | 1979 | midge | NC_011639 |
| Kolente virus (KOLEV) | *Ledantevirus kolente* | *Ledantevirus* | Guinea | 1985 | tick | NC_025342 |
| Kumasi rhabdovirus (KRV) | *Ledantevirus kumasi* | *Ledantevirus* | Ghana | 2011 | bat (*Eidolon helvum*) | NC_028236 |
| Le Dantec virus (LDV) | *Ledantevirus ledantec* | \| *Ledantevirus* \| \| --- \| | Senegal | \| 1965 \| \| --- \| | human | NC_034443 |
| Nkolbisson virus (NKOV) | *Ledantevirus nkolbisson* | *Ledantevirus* | Cameroon | 1965 | mosquitoe | NC_034539 |
| Barur virus (BARV) | *Ledantevirus barur* | \| *Ledantevirus* \| \| --- \| | India | \| 1962 \| \| --- \| | rat | NC_034535 |
| Fikirini virus (FKRV) | *Ledantevirus fikirini* | \| *Ledantevirus* \| \| --- \| | Kenya | \| 2011 \| \| --- \| | bat (*Hipposideros commersoni*) | NC_025341 |
| Fukuoka virus (FUKV) | *Ledantevirus fukuoka* | \| *Ledantevirus* \| \| --- \| | Japan | \| 1982 \| \| --- \| | midge | NC_034454 |
| Kern Canyon virus (KCV) | *Ledantevirus kern* | *Ledantevirus* | USA | 1968 | bat (*Myotis yumanensis*) | NC_034451 |
| Keuraliba virus (KEUV) | *Keuraliba virus* | *Ledantevirus* | Senegal | 1968 | gerbil | NC_034540 |
| \| 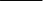Mount Elgon bat virus (MEBV) \| \| --- \| | \| *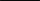Ledantevirus elgon* \| \| --- \| | \| *Ledantevirus* \| \| --- \| | Kenya | \| 1964 \| \| --- \| | \| bat (*Rhinolophus hilderbrandtii eloquens*) \| \| --- \| | \| NC_034545 \| \| --- \| |
| Nishimuro virus (NISV) | *Ledantevirus nishimuro* | *Ledantevirus* | Japan | 1962 | wild boar | AB609604 |
| Oita virus (OITAV) | *Ledantevirus oita* | \| *Ledantevirus* \| \| --- \| | Japan | \| 1972 \| \| --- \| | bat (*Rhinolophus cornutus*) | NC_034548 |
| Wuhan louse fly virus 5 (WLFV5) | *Ledantevirus wuhan* | *Ledantevirus* | China | 2013 | fly | NC_031301 |
| \| 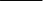Yongjia tick virus 2 (YTV2) \| \| --- \| | \| *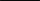Ledantevirus yongjia* \| \| --- \| | *Ledantevirus* | China | 2012 | tick | NC_031305 |
| Taiwan bat lyssavirus (TWBLV) | *Lyssavirus formosa* | *Lyssavirus* | Taiwan | 2016 | bat (*Pipistrellus abramus)* | MF472710 |
| Aravan virus (ARAV) | *Lyssavirus aravan* | *Lyssavirus* | Kyrgyzstan | 1991 | bat (*Myotis blythii*) | NC_020808 |
| Australian bat lyssavirus (ABLV) | *Lyssavirus australis* | *Lyssavirus* | Australia | 1996 | bat (*Saccolaimus flaviventris*) | NC_003243 |
| Bokeloh bat lyssavirus (BBLV) | *Lyssavirus bokeloh* | *Lyssavirus* | Germany | 2010 | bat (*Myotis nattererii*) | NC_025251 |
| Duvenhage virus (DUVV) | *Lyssavirus duvenhage* | *Lyssavirus* | South Africa | 1971 | human | NC_020810 |
| European bat lyssavirus 1 (EBLV1) | *Lyssavirus hamburg* | *Lyssavirus* | Germany | 1968 | bat (*Eptesicus serotinus*) | EF157976 |
| European bat lyssavirus 2 (EBLV2) | *Lyssavirus helsinki* | *Lyssavirus* | United Kingdom | 2016 | bat (*Myotis daubentonii*) | KY688156 |
| Gannoruwa bat lyssavirus (GBLV) | *Lyssavirus gannoruwa* | \| *Lyssavirus* \| \| --- \| | Sri Lanka | \| 2015 \| \| --- \| | \| bat (*Pteropus giganteus*) \| \| --- \| | \| NC_031988 \| \| --- \| |
| Ikoma lyssavirus (IKOV) | *Lyssavirus ikoma* | *Lyssavirus* | Tanzania | 2009 | African civet | NC_018629 |
| Irkut virus (IRKV) | *Lyssavirus irkut* | *Lyssavirus* | Russia | 2002 | bat (*Murina leucogaster*) | NC_020809 |
| Khujand virus (KHUV) | *Lyssavirus khujand* | *Lyssavirus* | Tajikstan | 2001 | bat (*Myotis mystacinus*) | NC_025385 |
| Lagos bat virus (LBV) | *Lyssavirus lagos* | *Lyssavirus* | Ghana | 2015 | bat (*Eidolon helvum*) | LN849915 |
| Lleida bat lyssavirus (LLEBV) | *Lyssavirus lleida* | \| *Lyssavirus* \| \| --- \| | Spain | 2011 | \| bat (*Miniopterus schreibersii*) \| \| --- \| | \| NC_031955 \| \| --- \| |
| Mokola virus (MOKV) | *Lyssavirus mokola* | \| *Lyssavirus* \| \| --- \| | South Africa | \| 1996 \| \| --- \| | feline | KF155008 |
| Rabies virus (RABV) | *Lyssavirus rabies* | \| *Lyssavirus* \| \| --- \| | France | \| 1983 \| \| --- \| | human | EU293121 |
| Shimoni bat virus (SHIBV) | *Lyssavirus shimoni* | \| *Lyssavirus* \| \| --- \| | Kenya | \| 2009 \| \| --- \| | bat (*Hipposideros commersoni*) | NC_025365 |
| West Caucasian bat virus (WCBV) | *Lyssavirus caucasicus* | **   \| *Lyssavirus* \| \| --- \| | Russia | \| 2001 \| \| --- \| | bat (*Miniopterus schreibersii*) | NC_025377 |
| Moussa virus (MOUV) | *Mousrhavirus moussa* | \| *Mousrhavirus* \| \| --- \| | Cote d'Ivoire | \| 2004 \| \| --- \| | mosquitoe | NC_025359 |
| Eel virus European X (EVEX) | *Perhabdovirus anguilla* | \| *Perhabdovirus* \| \| --- \| | Denmark | \| 1986 \| \| --- \| | fish | KC608035 |
| Perch rhabdovirus (PRV) | *Perhabdovirus perca* | *Perhabdovirus* | France | 1981 | fish | NC_020803 |
| Drosophila affinis sigmavirus (DAffSV) | *Sigmavirus affinis* | \| *Sigmavirus* \| \| --- \| | USA | \| 2007 \| \| --- \| | fly | KR822811 |
| Drosophila melanogaster sigmavirus (DMelSV) | *Sigmavirus melanogaster* | \| *Sigmavirus* \| \| --- \| | USA | \| unknown \| \| --- \| | fly | JX403934 |
| Drosophila obscura sigmavirus (DObSV) | *Sigmavirus obscura* | *Sigmavirus* | United Kingdom | 2007 | fly | NC_022580 |
| Carp sprivivirus (CSV) | *Sprivivirus cyprinus* | *Sprivivirus* | China | 2016 | fish | KY475636 |
| Grass carp rhabdovirus (GrCRV) | *Sprivivirus esox* | *Sprivivirus* | Germany | 1982 | fish | NC_025376 |
| Pike fry rhabdovirus (PFRV) | *Sprivivirus esox* | *Sprivivirus* | France | 1972 | fish | NC_025356 |
| \| Tench rhabdovirus (TenRV) \| \| --- \| | \| *Sprivivirus esox* \| \| --- \| | \| *Sprivivirus* \| \| --- \| | Germany | \| 1982 \| \| --- \| | fish | NC_025371 |
| Sena Madureira virus (SMV) | *Sripuvirus madureira* | \| *Sripuvirus* \| \| --- \| | Brazil | \| 1976 \| \| --- \| | lizard | NC_034529 |
| Sripur virus (SRIV) | *Sripuvirus sripur* | \| *Sripuvirus* \| \| --- \| | India | 1973 | \| sandfly \| \| --- \| | \| NC_034542 \| \| --- \| |
| Almpiwar virus (ALMV) | *Sripuvirus almpiwar* | *Sripuvirus* | Australia | 1966 | lizard | NC_025391 |
| \| Chaco virus (CHOV) \| \| --- \| | \| *Sripuvirus chaco* \| \| --- \| | \| *Sripuvirus* \| \| --- \| | Brazil | \| 1962 \| \| --- \| | lizard | NC_034550 |
| Niakha virus (NIAV) | *Sripuvirus niakha* | \| *Sripuvirus* \| \| --- \| | Senegal | \| 1992 \| \| --- \| | sandfly | NC_025405 |
| Oak Vale virus (OVV) | *Sunrhavirus oakvale* | *Sunrhavirus* | Australia | 1982 | mosquitoe | NC_025399 |
| Ekpoma virus 2 (EKV2) | *Tibrovirus betaekpoma* | *Tibrovirus* | Nigeria | 2011 | human | KP324828 |
| \| Bas-Congo virus (BASV) \| \| --- \| | \| *Tibrovirus congo* \| \| --- \| | \| *Tibrovirus* \| \| --- \| | Democratic Republic of the Congo | \| 2009 \| \| --- \| | human | JX297815 |
| Coastal Plains virus (CPV) | *Tibrovirus coastal* | *Tibrovirus* | Australia | 1981 | bovine | NC_025397 |
| Ekpoma virus (EKV) | *Unclassified* | \| *Tibrovirus* \| \| --- \| | China | \| 2017 \| \| --- \| | human | MF079256 |
| Ekpoma virus 1 (EKV1) | *Tibrovirus alphaekpoma* | \| *Tibrovirus* \| \| --- \| | Nigeria | 2011 | \| human \| \| --- \| | \| KP324827 \| \| --- \| |
| Sweetwater Branch virus (SWBV) | *Tibrovirus coastal* | *Tibrovirus* | USA | 1982 | midge | NC_034546 |
| Tibrogargan virus (TIBV) | *Tibrovirus tibrogargan* | \| *Tibrovirus* \| \| --- \| | Australia | \| 1976 \| \| --- \| | \| midge \| \| --- \| | \| NC_020804 \| \| --- \| |
| Durham virus (DURV) | *Tupavirus durham* | \| *Tupavirus* \| \| --- \| | USA | 2005 | \| bird \| \| --- \| | \| FJ952155 \| \| --- \| |
| Klamath virus (KLAV) | *Tupavirus klamath* | *Tupavirus* | USA | 1962 | vole | NC_034549 |
| Tupaia virus (TUPV) | *Tupavirus tupaia* | *Tupavirus* | Thailand | unknown | tree shrew | NC_007020 |
| American bat vesiculovirus (ABVV) | *Vesiculovirus eptesicus* | *Vesiculovirus* | USA | 2008 | bat (*Eptesicus fuscus*) | NC_022755 |
| Carajas virus (CARV) | *Vesiculovirus carajas* | *Vesiculovirus* | Brazil | 1983 | sandfly | KM205015 |
| Chandipura virus (CHPV) | *Vesiculovirus chandipura* | \| *Vesiculovirus* \| \| --- \| | India | 2004 | \| human \| \| --- \| | \| NC_020805 \| \| --- \| |
| Cocal virus (COCV) | *Vesiculovirus cocal* | \| *Vesiculovirus* \| \| --- \| | Trinidad | \| 1961 \| \| --- \| | rodent | EU373657 |
| Isfahan virus (ISFV) | *Vesiculovirus isfahan* | \| *Vesiculovirus* \| \| --- \| | Iran | 1975 | \| sandfly \| \| --- \| | \| NC_020806 \| \| --- \| |
| Jurona virus (JURV) | *Vesiculovirus jurona* | *Vesiculovirus* | Brazil | 1962 | human | NC_025392 |
| Malpais Spring virus (MSPV) | *Vesiculovirus malpais* | *Vesiculovirus* | USA | 1985 | mosquitoe | NC_025364 |
| Maraba virus (MARAV) | *Vesiculovirus maraba* | \| *Vesiculovirus* \| \| --- \| | Brazil | \| 1983 \| \| --- \| | sandfly | NC_025255 |
| Morreton virus (MORV) | *Vesiculovirus morreton* | *Vesiculovirus* | Colombia | 1986 | sandfly | NC_034508 |
| Perinet virus (PERV) | *Vesiculovirus perinet* | \| *Vesiculovirus* \| \| --- \| | Madagascar | \| 2008 \| \| --- \| | mosquito | NC_025394 |
| Piry virus (PIRYV) | *Vesiculovirus piry* | \| *Vesiculovirus* \| \| --- \| | Brazil | \| 1960 \| \| --- \| | Philander opossum | KU178986 |
| Radi virus (RADV) | *Vesiculovirus radi* | *Vesiculovirus* | Italy | 1982 | sandfly | KM205024 |
| Vesicular stomatitis Alagoas virus (VSAV) | *Vesiculovirus indiana* | *Vesiculovirus* | USA | 1998 | horse | NC_001560 |
| Vesicular stomatitis New Jersey virus (VSNJV) | *Vesiculovirus newjersey* | *Vesiculovirus* | Honduras | 1984 | bovine | NC_024473 |
| VS Alagoas virus (VSAV) | *Vesiculovirus alagoas* | *Vesiculovirus* | Brazil | 1964 | mule | NC_025353 |
| Yug Bogdanovac virus (YBV) | *Vesiculovirus bogdanovac* | *Vesiculovirus* | Serbia | 2011 | Vero-E6 cells | NC_025378 |
