# Supplementary material for: Broad geographical circulation of a novel vesiculovirus in bats in the Mediterranean region
Source: PLoS Negl Trop Dis. 2025 Jun 12;19(6):e0013172. doi: 10.1371/journal.pntd.0013172 (PMC12193708; doi:10.1371/journal.pntd.0013172)
Supplement: S3 Table — (DOCX) [file pntd.0013172.s007.docx]

**Table S3.** Description of the primers tested during the validation step of the different pan-rhabdovirus PCR systems.

| **PCR system** | **Type** | **Primer** | **Sequence (5'-3')** | **Length (nt)** | **Reference position** | **Amplicon length (bp)^b^** | **Reference** |
| --- | --- | --- | --- | --- | --- | --- | --- |
| Screen-rhabdo qPCR_1 | SYBR Green-qPCR | F1_Rhabdovirus | ATWGGNYTNAARSSIAARGA | 20 | 1633-1652^a^ | 155 | This study |
|  |  | 2000R1_Rhabdovirus | ADRTYRTCNGYCATIGTIA | 19 | 1769-1787^a^ |  |  |
| Screen-rhabdo qPCR_2 | SYBR Green-qPCR | 2100F2_Rhabdovirus | TNGAYTAYGANAARTGGAAIAA | 22 | 1871-1892^a^ | 121 | This study |
|  |  | 2220R2_Rhabdovirus | TTYTBAAANAAITYRTGIG | 19 | 1973-1991^a^ |  |  |
| Screen-rhabdo qPCR_3 | SYBR Green-qPCR | 2340F3_Rhabdovirus | GARGGNYTNMGDCARAAIGGITGG | 24 | 2098-2121^a^ | 119 | This study |
|  |  | R1_Rhabdovirus | RYYTGRTTRTCNCCYTGIGC | 20 | 2188-2207^a^ |  |  |
| Screen-rhabdo qPCR_4 | SYBR Green-qPCR | F2_Rhabdovirus-M | GAYTAYGANAARTGGAAYAAYYAYCA | 26 | 1873-1898^a^ | 239 | This study |
|  |  | R2_Rhabdovirus | TGYCKNARNCCYTCYARNCCICC | 23 | 2089-2111^a^ |  |  |
| Pan-rhabdo RT-nqPCR | Conventional PCR (Nest first round) | F1_Rhabdovirus | ATWGGNYTNAARSSIAARGA | 20 | 1633-1652^a^ | 575 | This study |
|  |  | R1_Rhabdovirus | RYYTGRTTRTCNCCYTGIGC | 20 | 2188-2207^a^ |  |  |
|  | SYBR Green-qPCR/ Conventional PCR (Nest second round) | F2_Rhabdovirus-M | GAYTAYGANAARTGGAAYAAYYAYCA | 26 | 1873-1898^a^ | 239 |  |
|  |  | R2_Rhabdovirus | TGYCKNARNCCYTCYARNCCICC | 23 | 2089-2111^a^ |  |  |
| Rhabdo-screening nest conventional PCR_1 | Conventional PCR (Nest first round) | DimLis1F | GGKMGRTTYTTYKCHYTDATG | 21 | 1650–1670^b^ | 466 | [1] |
|  |  | DimLis1R | CARAARGGNTGGASYNTHBT | 20 | 2097-2116^b^ |  |  |
|  | Conventional PCR (Nest second round) | DimLis2F | YTNTTYVANGSVYTRACNATG | 21 | 1734–1754^b^ | 150 |  |
|  |  | DimLis2R | TGGAAYAAYCAYCARMGRHWD | 21 | 1854–1874^b^ |  |  |
| Rhabdo-screening nest conventional PCR_2 | Conventional PCR (Nest first round) | PVO3 | CCADMCBTTTTGYCKYARRCCTTC | 24 | 1655–1678^b^ | 458 | [2] |
|  |  | PVO4 | RAAGGYAGRTTTTTYKCDYTRATG | 24 | 2090-2113^b^ |  |  |
|  | Conventional PCR (Nest second round) | PVO3 | CCADMCBTTTTGYCKYARRCCTTC | 24 | 1655–1678^b^ | 260 |  |
|  |  | PVOnstF | AARTGGAAYAAYCAYCARMG | 20 | 1896-1915^b^ |  |  |

R=A/G, Y=C/T, M=A/C, K=G/T, S=G/C, W=A/T, H=A/T/C, B=G/T/C, V=G/A/C, D=G/A/T, N=A/T/C/G and I (hypoxanthine).

^a^ According to the Drosophila obscura sigmavirus (DObSV) L gene nucleotide sequence (GenBank accession number NC022580).

^b^ According to the rabies virus (RABV, CVS strain) L gene nucleotide sequence (GenBank accession number GQ918139).

**References**

1. Aznar-Lopez C, Vazquez-Moron S, Marston DA, Juste J, Ibanez C, Berciano JM, et al. Detection of rhabdovirus viral RNA in oropharyngeal swabs and ectoparasites of Spanish bats. Journal of General Virology. 2013;94: 69–75. doi:10.1099/vir.0.046490-0.

2. Wray AK, Olival KJ, Morán D, Lopez MR, Alvarez D, Navarrete-Macias I, et al. Viral Diversity, Prey Preference, and Bartonella Prevalence in Desmodus rotundus in Guatemala. EcoHealth. 2016;13: 761–774. doi:10.1007/s10393-016-1183-z.
