# Supplementary material for: Broad geographical circulation of a novel vesiculovirus in bats in the Mediterranean region
Source: PLoS Negl Trop Dis. 2025 Jun 12;19(6):e0013172. doi: 10.1371/journal.pntd.0013172 (PMC12193708; doi:10.1371/journal.pntd.0013172)
Supplement: S4 Table — (DOCX) [file pntd.0013172.s008.docx]

**Table S4.** Description of the samples tested (rhabdovirus isolates and field samples) and the results obtained after the comparative evaluation of the different primers and qPCR systems.

| **Virus isolate** | **Strain** | **Species** | **Genus** | **Origin** | **Sample type** | **PCR results from different assay** | | | | | | |
| --- | --- | --- | --- | --- | --- | --- | --- | --- | --- | --- | --- | --- |
|  |  |  |  |  |  | **Rhabdo-screening qPCR_1** | **Rhabdo-screening qPCR_2** | **Rhabdo-screening qPCR_3** | **Rhabdo-screening qPCR_4** | **Pan-rhabdo-RT-nqPCR** | **Rhabdo-screening nest conventional PCR_1**^e^ | **Rhabdo-screening nest conventional PCR_2**^f^ |
| Sandjimba virus (SJAV) | DakAnB 373d (0408RCA) | *Sunrhavirus sandjimba* | *Sunrhavirus* | Bird (*Acrocephalus schoenbaeus*) | Brain (mouse)^c^ | Negative | Negative | Negative | Positive | Positive | ND | ND |
| Piry virus (PIRV) | BeAn 24232 (0413BRE) | *Vesiculovirus piry* | *Vesiculovirus* | Gray four-eyed opossums (*Philander opossum*) | Brain (mouse)^c^ | Positive | Positive | Positive | Positive | Positive | Negative | Positive |
| Jurona virus (JURV) | BeAr 40578 (0414BRE) | *Vesiculovirus jurona* | *Vesiculovirus* | Human | Brain (mouse)^c^ | Positive | Positive | Positive | Positive | Positive | ND | ND |
| Nkolbisson virus (NKOV) | Ar YM 31/65 (0425CAM) | *Ledantevirus nkolbisson* | *Ledantevirus* | Mosquito (*Eretmapodites leucopous*) | Brain (mouse)^c^ | Negative | Negative | Negative | Positive | Positive | ND | ND |
| Keuraliba virus (KEUV) | DakAnD 5314 (9715SEN) | *Ledantevirus keuraliba* | *Ledantevirus* | Gerbil (*Tatera kempi*) | Brain (mouse)^c^ | Negative | Positive | Positive | Positive | Positive | ND | ND |
| Bovine ephemeral fever virus (BEFV) | 7635MAY | *Ephemeroviru febris* | *Ephemerovirus* | Bovine | Blood^d^ | Negative | Negative | Negative | Negative | Positive | ND | ND |
| Bovine ephemeral fever virus (BEFV) | 7645MAY | *Ephemerovirus febris* | *Ephemerovirus* | Bovine | Blood^d^ | Negative | Positive | Negative | Positive | Positive | ND | ND |
| Vesicular stomatitis New Jersey virus (VSNJV) | VSV NJ-O (05004FRA) | *Vesiculovirus newjersey* | *Vesiculovirus* | Bovine | Brain (mouse)^c^ | ND^b^ | ND | ND | ND | Positive | Negative | Negative |
| Mediterranean bat virus (MBV) | A09061 | *Vesiculovirus mediterranean* | *Vesiculovirus* | Bat (*Rhinolophus ferrumequinum*) | Blood^d^ | ND | ND | ND | Negative | Positive | Negative | Positive |
| Mediterranean bat virus (MBV | A09097 | *Vesiculovirus mediterranean* | *Vesiculovirus* | Bat (*Rhinolophus ferrumequinum*) | Blood^d^ | ND | ND | ND | Negative | Positive | Negative | Positive |
| NA^a^ | E08157 | NA | NA | Bat (*Rousettus aegyptiacus*) | Blood^d^ | ND | ND | ND | ND | Negative | Negative | Negative |
| NA | E08159 | NA | NA | Bat (*Rousettus aegyptiacus*) | Blood^d^ | ND | ND | ND | ND | Negative | Negative | Negative |
| NA | E08195 | NA | NA | Bat (*Rousettus aegyptiacus*) | Blood^d^ | ND | ND | ND | ND | Negative | Negative | Negative |
| NA | A09027 | NA | NA | Bat (*Miniopterus schreibersii*) | Blood^d^ | ND | ND | ND | ND | Negative | Negative | Negative |
| NA | A09037 | NA | NA | Bat (*Miniopterus schreibersii*) | Blood^d^ | ND | ND | ND | ND | Negative | Negative | Negative |
| NA | A09039 | NA | NA | Bat (*Miniopterus schreibersii*) | Blood^d^ | ND | ND | ND | ND | Negative | Negative | Negative |
| NA | D0T039 | NA | NA | Bat (*Micropteropus pusillus* ) | Oral swab^d^ | ND | ND | ND | ND | Negative | Negative | Negative |
| NA | DOT042 | NA | NA | Bat (*Micropteropus pusillus* ) | Oral swab^d^ | ND | ND | ND | ND | Negative | Negative | Negative |
| NA | DOT061 | NA | NA | Bat (*Micropteropus pusillus* ) | Oral swab^d^ | ND | ND | ND | ND | Negative | Negative | Negative |
| NA | KAB11 | NA | NA | Bat (*Micropteropus pusillus* ) | Oral swab^d^ | ND | ND | ND | ND | Negative | Negative | Negative |

^a^ NA: Not applicable (negative sample).

^b^ ND: Not done.

^c^ Laboratory sample.

^d^ Field sample.

^e^ Nested conventional PCR published by [1].

^f^ Nested conventional PCR published by [2].

**References**

1. Aznar-Lopez C, Vazquez-Moron S, Marston DA, Juste J, Ibanez C, Berciano JM, et al. Detection of rhabdovirus viral RNA in oropharyngeal swabs and ectoparasites of Spanish bats. Journal of General Virology. 2013;94: 69–75. doi:10.1099/vir.0.046490-0.

2. Wray AK, Olival KJ, Morán D, Lopez MR, Alvarez D, Navarrete-Macias I, et al. Viral Diversity, Prey Preference, and Bartonella Prevalence in Desmodus rotundus in Guatemala. EcoHealth. 2016;13: 761–774. doi:10.1007/s10393-016-1183-z.
