# Supplementary material for: Broad geographical circulation of a novel vesiculovirus in bats in the Mediterranean region
Source: PLoS Negl Trop Dis. 2025 Jun 12;19(6):e0013172. doi: 10.1371/journal.pntd.0013172 (PMC12193708; doi:10.1371/journal.pntd.0013172)
Supplement: S5 Table — (DOCX) [file pntd.0013172.s009.docx]

**Table S5.** Results of pan-rhabdo RT-nqPCR detection with a panel of representative members of different genera of the family *Rhabdoviridae*.

| **Virus (acronym)** | **Isolate** | **Species** | **Genus** | **Origin** | **Country** | **Type of sample^a^** | **Pan-rhabdo RT-nqPCR results** |
| --- | --- | --- | --- | --- | --- | --- | --- |
| Rabies virus (RABV) | 201020958 | *Rabies lyssavirus* | *Lyssavirus* | Dog (*Canis lupus familiaris*) | Spain | RNA (mouse brain) | Positive |
| Rabies virus (RABV) | CVS 27 11-14 | *Rabies lyssavirus* | *Lyssavirus* | Lab strain | - | RNA (mouse brain) | Positive |
| Rabies virus (RABV) | d1018516 | *Rabies lyssavirus* | *Lyssavirus* | Dog (*Canis lupus familiaris*) | Cambodia | RNA (dog brain) | Positive |
| Rabies virus (RABV) | d1019384 | *Rabies lyssavirus* | *Lyssavirus* | Dog (*Canis lupus familiaris*) | Cambodia | RNA (dog brain) | Positive |
| Rabies virus (RABV) | d1019390 | *Rabies lyssavirus* | *Lyssavirus* | Dog (*Canis lupus familiaris*) | Cambodia | RNA (dog brain) | Positive |
| Rabies virus (RABV) | d1024470 | *Rabies lyssavirus* | *Lyssavirus* | Dog (*Canis lupus familiaris*) | Cambodia | RNA (dog brain) | Positive |
| Rabies virus (RABV) | d1028271 | *Rabies lyssavirus* | *Lyssavirus* | Dog (*Canis lupus familiaris*) | Cambodia | RNA (dog brain) | Positive |
| Rabies virus (RABV) | GS7 | *Rabies lyssavirus* | *Lyssavirus* | Fox (Vulpes vulpes) | France | RNA (fox brain) | Positive |
| Australian bat lyssavirus (ABLV) | 06-17 | *Lyssavirus australis* | *Lyssavirus* | Bat (*Pteropus alecto*) | Australia | RNA (mouse brain) | Positive |
| Bokeloh bat lyssavirus (BBLV) | 127900 | *Lyssavirus bokeloh* | *Lyssavirus* | Bat (*Myotis nattereri*) | France | RNA (mouse brain) | Positive |
| European bat lyssavirus 1 (EBLV-1) | 18002FRA | *Lyssavirus hamburg* | *Lyssavirus* | Bat (*Eptesicus serotinus*) | France | RNA (bat brain) | Positive |
| European bat lyssavirus 1 (EBLV-1) | 18003FRA | *Lyssavirus hamburg* | *Lyssavirus* | Bat (*Eptesicus serotinus*) | France | RNA (bat brain) | Positive |
| European bat lyssavirus 1 (EBLV-1) | 19011FRA | *Lyssavirus hamburg* | *Lyssavirus* | Bat (*Eptesicus serotinus*) | France | RNA (bat brain) | Positive |
| European bat lyssavirus 1 (EBLV-1) | 123008 | *Lyssavirus hamburg* | *Lyssavirus* | Bat (*Eptesicus serotinus*) | France | RNA (mouse brain) | Positive |
| European bat lyssavirus 1 (EBLV-1) | 18002FRA | *Lyssavirus hamburg* | *Lyssavirus* | Bat (*Eptesicus serotinus*) | France | RNA (bat brain) | Positive |
| European bat lyssavirus 1 (EBLV-1) | 18003FRA | *Lyssavirus hamburg* | *Lyssavirus* | Bat (*Eptesicus serotinus*) | France | RNA (bat brain) | Positive |
| European bat lyssavirus 2 (EBLV-2) | RV1787 | *Lyssavirus helsinki* | *Lyssavirus* | Bat (*Myotis daubentonii*) | United Kingdom | RNA (mouse brain) | Positive |
| Nkolbisson virus (NKOV) | YM 31-65 (0425CAM) | *Ledantevirus nkolbisson* | *Ledantevirus* | Mosquito (*Eretmapodites leucopous*) | Cameroon | RNA (mouse brain) | Positive |
| Le Dantec virus (LDV) | DakHD763 (9006SEN) | *Ledantevirus ledantec* | *Ledantevirus* | Human | Senegal | RNA (mouse brain) | Positive |
| Keuraliba virus (KEUV) | DakAnD5314 (9715SEN) | *Ledantevirus keuraliba* | *Ledantevirus* | Gerbil (*Tatera kempi*) | Senegal | RNA (mouse brain) | Positive |
| Kolente virus (KOLEV) | DakAr K7292 (11034GUI) | *Ledantevirus kolente* | *Ledantevirus* | Bat (*Hipposideros sp.*) | Guinea | RNA (mouse brain) | Positive |
| Vesicular stomatitis Indiana virus (VSIV) | Indiana (05003FRA) | *Vesiculovirus indiana* | *Vesiculovirus* | Horse | USA | RNA (cell culture) | Positive |
| Vesicular stomatitis New Jersey virus (VSNJV) | New Jersey (05004FRA) | *Vesiculovirus newjersey* | *Vesiculovirus* | Bovine | USA | RNA (cell culture) | Positive |
| Cocal virus (COCV) | TRVL 40233 (0412TRI) | *Vesiculovirus cocal* | *Vesiculovirus* | Mite (*Gigantolaelaps sp*.) | Trinidad | RNA (mouse brain) | Positive |
| Piry virus (PIRYV) | BeAn 2423 (0413BRE) | *Vesiculovirus piry* | *Vesiculovirus* | Gray four-eyed opossums (*Philander opossum*) | Brazil | RNA (mouse brain) | Positive |
| Jurona virus (JURV) | BeAr 40578 (0414BRE) | *Vesiculovirus jurona* | *Vesiculovirus* | Human | Brazil | RNA (mouse brain) | Positive |
| Perinet virus (PERV) | DAkAr Mg802 (9714MAD) | *Vesiculovirus perinet* | *Vesiculovirus* | Dipterans | Madagascar | RNA (mouse brain) | Positive |
| Qiongzhong bat virus (QZBV) | 1127 | *Vesiculovirus rhinolophus* | *Vesiculovirus* | Bat (*Rhinolophus affinis*) | China | RNA (bat brain) | Positive |
| Yinshui bat virus (YSBV) | 1017 | *Vesiculovirus yinshui* | *Vesiculovirus* | Bat (*Rhinolophus sinica*) | China | RNA (bat brain) | Positive |
| Yinshui bat virus (YSBV) | D170001 | *Vesiculovirus yinshui* | *Vesiculovirus* | Bat (*Rhinolophus sinica*) | China | RNA (bat brain) | Positive |
| Yinshui bat virus (YSBV) | D170022 | *Vesiculovirus yinshui* | *Vesiculovirus* | Bat (*Rhinolophus sinica*) | China | RNA (bat brain) | Positive |
| Yinshui bat virus (YSBV) | D170190 | *Vesiculovirus yinshui* | *Vesiculovirus* | Bat (*Rhinolophus sinica*) | China | RNA (bat brain) | Positive |
| Obodhiang virus (OBOV) | SudAr 1154-64 (0426SUD) | *Ephemerovirus obodhiang* | *Ephemerovirus* | Mosquito (*Mansonia uniformis*) | Sudan | RNA (mouse brain) | Positive |
| Kotonkan viruss (KOTV) | IbAr 23380 (9145NIG) | *Ephemerovirus kotonkan* | *Ephemerovirus* | Midge (*Culicoides sp.*) | Nigeria | RNA (mouse brain) | Positive |
| Adelaide River virus (ARV) | DPP 61 (ARV, B150) | *Ephemerovirus adelaide* | *Ephemerovirus* | Bovine | Australia | RNA (mouse brain) | Positive |
| Kimberley virus (KIMV) | CS 368 (KIMV, B16) | *Ephemerovirus kimberley* | *Ephemerovirus* | Bovine | Australia | RNA (mouse brain) | Positive |
| Bovine ephemeral fever virus (BEFV) | 7635 | *Ephemerovirus febris* | *Ephemerovirus* | Bovine | Mayotte, France | RNA (bovine blood) | Positive |
| Bovine ephemeral fever virus (BEFV) | 7639 | *Ephemerovirus febris* | *Ephemerovirus* | Bovine | Mayotte, France | RNA (bovine blood) | Positive |
| Bovine ephemeral fever virus (BEFV) | 7641 | *Ephemerovirus febris* | *Ephemerovirus* | Bovine | Mayotte, France | RNA (bovine blood) | Positive |
| Bovine ephemeral fever virus (BEFV) | 7645 | *Ephemerovirus febris* | *Ephemerovirus* | Bovine | Mayotte, France | RNA (bovine blood) | Positive |
| Bovine ephemeral fever virus (BEFV) | 7620 | *Ephemerovirus febris* | *Ephemerovirus* | Bovine | Mayotte, France | RNA (bovine blood) | Positive |
| Porton virus (PORV) | S 1643 (0416MAL) | *Hapavirus porton* | *Hapavirus* | Dipteran (*Mansonia uniformis*) | Malaysia | RNA (mouse brain) | Positive |
| Mossuril virus (MOSV) | SA Ar 1995 (0418MOZ) | *Hapavirus mossuril* | *Hapavirus* | Mosquito (*Culex sitiens*) | Mozambique | RNA (mouse brain) | Positive |
| Kamese virus (KAMV) | MP 6186 (0419OUG) | *Hapavirus kamese* | *Hapavirus* | Mosquito (*Culex pruina*) | Uganda | RNA (mouse brain) | Positive |
| Bangoran virus (BGNV) | DakArB 2053 (0424RCA) | *Hapavirus bangoran* | *Hapavirus* | Dipteran (*Culex perfuscus*) | Central African Republic | RNA (mouse brain) | Positive |
| Landjia virus (LJAV) | DakAnB769d (9026RCA) | *Hapavirus landjia* | *Hapavirus* | Bird (*Riparia paludicola*) | Central African Republic | RNA (mouse brain) | Positive |
| Perch rhabdovirus (PRV) | 16-65 (19005FRA) | *Perhabdovirus perca* | *Perhabdovirus* | Perch (*Percidae*) | Belgium | RNA (cell culture) | Positive |
| Perch rhabdovirus (PRV) | P8350 (19003FRA) | *Perhabdovirus perca* | *Perhabdovirus* | Perch (*Percidae*) | France | RNA (cell culture) | Positive |
| Leman virus (LeRV) | 18-193 (19001FRA) | *Perhabdovirus leman* | *Perhabdovirus* | Perch (*Percidae*) | France | RNA (cell culture) | Positive |
| Lake trout rhabdovirus (LTRV) | 18-203 (19002FRA) | *Perhabdovirus trutta* | *Perhabdovirus* | Sea trout (*Percidae*) | France | RNA (cell culture) | Positive |
| Lake trout rhabdovirus (LTRV) | R6146 (19004FRA) | *Perhabdovirus trutta* | *Perhabdovirus* | Sea trout (*Percidae*) | France | RNA (cell culture) | Positive |
| Drosophila melanogaster sigmavirus (DMelSV) | CQI-0 | *Sigmavirus melanogaster* | *Sigmavirus* | Insect | - | RNA (cell culture) | Positive |
| Sandjimba virus (SJAV) | DakAnB 373d (0408RCA) | *Sunrhavirus sandjimba* | *Sunrhavirus* | Bird (*Acrocephalus schoenbaeus*) | Central African Republic | RNA (mouse brain) | Positive |
| Nasoule virus (NASV) | DakAnB 4289a (0410RCA) | *Sunrhavirus nasoule* | *Sunrhavirus* | Bird (*Andropadus virens*) | Central African Republic | RNA (mouse brain) | Positive |
| Boteke virus (BOTV) | DakAnB 1077 (0417RCA) | *Sunrhavirus boteke* | *Sunrhavirus* | Dipteran (*Coquillettidia maculipennis*) | Central African Republic | RNA (mouse brain) | Positive |
| Garba virus (GARV) | DakAnB 439a (0422RCA) | *Sunrhavirus garba* | *Sunrhavirus* | Bird (*Corythornis cristata*) | Central African Republic | RNA (mouse brain) | Positive |
| Burg el Arab virus (BEAV) | UAR An 3782-62 (09023EGY) | *Sunrhavirus alexandria* | *Sunrhavirus* | Bird (*Sylvia curraca*) | Egypt | RNA (mouse brain) | Positive |
| Matariya virus (MTYV) | EgAn 1477-61 (09027EGY) | *Sunrhavirus matariya* | *Sunrhavirus* | Bird (*Sylvia curraca*) | Egypt | RNA (mouse brain) | Positive |
| Bimbo virus (BBOV) | DakAnB 1054d (9716RCA) | *Sunrhavirus bimbo* | *Sunrhavirus* | Bird (*Euplectes afra*) | Central African Republic | RNA (mouse brain) | Positive |
| Ouango virus (OUAV) | DakAnB 1582a (9718RCA) | *Sunrhavirus ouango* | *Sunrhavirus* | Bird (*Ploceus melanocephalus*) | Central African Republic | RNA (mouse brain) | Positive |

^a^ All samples are field specimens, except mouse brain and cell culture samples.
