# Supplementary material for: Broad geographical circulation of a novel vesiculovirus in bats in the Mediterranean region
Source: PLoS Negl Trop Dis. 2025 Jun 12;19(6):e0013172. doi: 10.1371/journal.pntd.0013172 (PMC12193708; doi:10.1371/journal.pntd.0013172)
Supplement: S6 Table — (DOCX) [file pntd.0013172.s010.docx]

**Table S6.** Specificity analysis of the pan-rhabdo RT-nqPCR on a panel of representative viruses other than rhabdoviruses and on a panel of non-infected samples.

| **Virus** | **Isolate** | **Species** | **Family** | **Genome^c^** | **Origin** | **Type of sample^d^** | **Pan-rhabdo RT-nqPCR results** |
| --- | --- | --- | --- | --- | --- | --- | --- |
| Measles virus | Edmonston Enders | *Morbillivirus hominis* | *Paramyxoviridae* | ssRNA (-) | Vaccine | RNA (lyophilized suspension for injection) | Negative |
| Mumps virus | Jeryl Lynn | *Orthorubulavirus parotitidis* | *Paramyxoviridae* | ssRNA (-) | Vaccine | RNA (lyophilized suspension for injection) | Negative |
| Bovine respiratory syncytial virus | Un^b^ | *Metapneumovirus avis* | *Pneumoviridae* | ssRNA (-) | Bovine | RNA (cell culture) | Negative |
| Human respiratory syncytial virus | Un | *Metapneumovirus hominis* | *Pneumoviridae* | ssRNA (-) | Human | RNA (cell culture) | Negative |
| Influenza A virus | H1N1, CQI-8 | *Influenza A virus* | *Orthomyxoviridae* | segmented ssRNA (-) | Bird | RNA (cell culture) | Negative |
| Influenza B virus | CQI-18 | *Influenza B virus* | *Orthomyxoviridae* | segmented ssRNA (-) | Bird | RNA (cell culture) | Negative |
| Rubella virus | Wistar | *Rubivirus rubellae* | *Matonaviridae* | ssRNA (+) | Vaccine | RNA (lyophilized suspension for injection) | Negative |
| Chikungunya virus | CQI-12 | *Chikungunya virus* | *Togaviridae* | ssRNA (+) | Mosquito | RNA (cell culture) | Negative |
| Dengue virus | CQI-14 | *Orthoflavivirus denguei* | *Flaviviridae* | ssRNA (+) | Mosquito | RNA (cell culture) | Negative |
| Zika virus | CQI-15 | *Orthoflavivirus zikaense* | *Flaviviridae* | ssRNA (+) | Mosquito | RNA (cell culture) | Negative |
| Enterovirus A71 | EV-A71 BrCr | *Enterovirus A* | *Picornaviridae* | ssRNA (+) | Un | RNA (cell culture) | Negative |
| Coxsackievirus B2 | CV-B2 Ohio-1 | *Enterovirus B* | *Picornaviridae* | ssRNA (+) | Un | RNA (cell culture) | Negative |
| Coxsackievirus A11 | CV-A11 Belgium-1 | *Enterovirus C* | *Picornaviridae* | ssRNA (+) | Un | RNA (cell culture) | Negative |
| Enterovirus D68 | EV-D68 Fermon | *Enterovirus D* | *Picornaviridae* | ssRNA (+) | Un | RNA (cell culture) | Negative |
| Rhinovirus | Rhinovirus (3) | *Rhinovirus sp.* | *Picornaviridae* | ssRNA (+) | Un | RNA (cell culture) | Negative |
| Vaccinia virus | CQI-1 | *Vaccinia virus* | *Poxviridae* | dsDNA | Vaccine | RNA (cell culture) | Negative |
| Varicella-zoster virus | CQI-3 | *Varicellovirus humanalpha3* | *Herpesviridae* | dsDNA | Un | RNA (cell culture) | Negative |
| Herpes simplex virus 2 | CQI-4 | *Simplexvirus humanalpha2* | *Herpesviridae* | dsDNA | Un | RNA (cell culture) | Negative |
| NA^a^ | Fox brain 12-15 | NA | NA | NA | Fox (*Vulpes vulpes*) | RNA (fox brain) | Negative |
| NA | Fox brain 38-12 | NA | NA | NA | Fox (*Vulpes vulpes*) | RNA (fox brain) | Negative |
| NA | Dog brain | NA | NA | NA | Dog (*Canis lupus familiaris*) | RNA (dog brain) | Negative |
| NA | FLG-R cell line | NA | NA | NA | Bat (*Eptesicus serotinus*) | RNA (cell line) | Negative |
| NA | FLG-ID cell line | NA | NA | NA | Bat (*Eptesicus serotinus*) | RNA (cell line) | Negative |
| NA | Tb1 cell line | NA | NA | NA | Bat (*Tadarida brasiliensis*) | RNA (cell line) | Negative |
| NA | Vero cell line | NA | NA | NA | African green monkey (*Chlorocebus aethiops*) | RNA (cell line) | Negative |

^a^ NA: Not applicable.

^b^ Un: Uknown.

^c^ ssRNA (-): single-stranded negative RNA, segmented ssRNA (-): segmented single-stranded negative RNA, ssRNA (+): single-stranded positive RNA, dsDNA: double stranded DNA.

^d^ All samples are lab specimens, except dog and fox brains.
