# Supplementary material for: Broad geographical circulation of a novel vesiculovirus in bats in the Mediterranean region
Source: PLoS Negl Trop Dis. 2025 Jun 12;19(6):e0013172. doi: 10.1371/journal.pntd.0013172 (PMC12193708; doi:10.1371/journal.pntd.0013172)
Supplement: S7 Table — (DOCX) [file pntd.0013172.s011.docx]

**Table S7.** Specific primers designed to complete the full-length genome sequences of the bat Mediterranean vesiculovirus (MBV) isolates.

| **Target sample** | **Primer** | **Sequence** | **Amplicon size (bp)** |
| --- | --- | --- | --- |
| 2012096^a^ | 2012096_N-terminal_F1 | 5' TTGTCCCAAAGTGTCTCGCG 3' | 560 |
|  | 2012096_N-terminal_R1 | 5' CATTAGAAGAGCCCGATACTCTGG 3' |  |
|  | 2012096_N-terminal_F2 | 5' TCTCGCGTCCCGACCACC 3' |  |
|  | 2012096_N-terminal_R2 | 5' GAGGAGGTATGTCGGTAGCC 3' |  |
|  | 2012096_M-G_F1 | 5' CCTGCAGACATAGTTAAGTGGGC 3' | 1300 |
|  | 2012096_M-G_R1 | 5' GTGTCCTCATACACTGTTTTACAGGG 3' |  |
|  | 2012096_M-G_F2 | 5' CAGATTCTCAGTCCGTTATGATCTGGC 3' |  |
|  | 2012096_M-G_R2 | 5' TCATCCACTCCCACGTGGTGTGGG 3' |  |
|  | 2012096_L-1_F1 | 5' GACTGGAAGGGCCCGGATGATC 3' | 1700 |
|  | 2012096_L-1_R1 | 5' TCTACCACCCTCATCAAATCAGG 3' |  |
|  | 2012096_L-1_F2 | 5' GCCTGGTTTGCATCATGGATTGG 3' |  |
|  | 2012096_L-1_R2 | 5' TGGGGTAACCTAAAAACTGGCC 3' |  |
|  | 2012096_L-2_F1 | 5' CCAAAATGAAGTCATACGTGATTC 3' | 1500 |
|  | 2012096_L-2_R1 | 5' CTGAGTGGACAAAACAAAAGGCC 3' |  |
|  | 2012096_L-2_F2 | 5' CCTATGGTCCATTGACCCCTTATTTCCC 3' |  |
|  | 2012096_L-2_R2 | 5' GCAGTGTCAGAAAATATCCAAATCGCAGG 3' |  |
|  | 2012096_L-3_F1 | 5' GGATGTGTACAACTCAACATTAACGG 3' | 500 |
|  | 2012096_L-3_R1 | 5' CCTGATTCATAAATCCCCCCTCC 3' |  |
|  | 2012096_L-3_F2 | 5' CGATTCAAAATATCGACTAAGATGG 3' |  |
|  | 2012096_L-3_R2 | 5' CCCCACAGAAGCAGATGGTACGG 3' |  |
| A09145^b^ | A09145_N-terminal_F1 | 5' TTGTCCCAAAGTGTCTCGCG 3' | 360 |
|  | A09145_N-terminal_R1 | 5' TGTCCATATTCTCCTCCGCG 3' |  |
|  | A09145_N-terminal_F2 | 5' TCCCGACCACCCATTGATTG 3' |  |
|  | A09145_N-terminal_R2 | 5' ATAGTTATGCCGAAGGATGTCC 3' |  |

^a^ Virus isolate from an oral swab sample collected from a Spanish bat.

^b^ Virus isolate from a blood sample collected from an Algerian bat.
