# Supplementary material for: Broad geographical circulation of a novel vesiculovirus in bats in the Mediterranean region
Source: PLoS Negl Trop Dis. 2025 Jun 12;19(6):e0013172. doi: 10.1371/journal.pntd.0013172 (PMC12193708; doi:10.1371/journal.pntd.0013172)
Supplement: S8 Table — The lyssavirus RABV was selected as an outlier. (DOCX) [file pntd.0013172.s012.docx]

**Table S8.** List of vesiculoviruses and sequence identification selected for phylogenetic analysis of Mediterraean bat vesiculovirus isolates. The lyssavirus RABV was selected as an outlier.

| **Virus (acronym)** | **Genus** | **Location of first isolation** | **Year of isolation** | **Host species** | **GenBank accession nb.** |
| --- | --- | --- | --- | --- | --- |
| Rabies lyssavirus (RABV) | *Lyssavirus* | France | 1983 | Human/Bat | EU293121 |
| Piry vesiculovirus(PIRV) | *Vesiculovirus* | Brazil | 1960 | Gray four-eyed opossums (*Philander opossum*) | KU178986 |
| Jurona vesiculovirus (JURV) | *Vesiculovirus* | Brazil | 1962 | Human | NC025392 |
| Carajas vesiculovirus (CARV) | *Vesiculovirus* | Brazil | 1983 | Sandfly (*Lutzomyia sp.*) | KM205015 |
| Maraba vesiculovirus (MARAV) | *Vesiculovirus* | Brazil | 1983 | Sandfly | NC025255 |
| VS Alagoas vesiculovirus (VSAV) | *Vesiculovirus* | Brazil | 1964 | Mule | NC025353 |
| Jinghong bat vesiculovirus (JHBV) | *Vesiculovirus* | China | 2011 | Bat (*Rhinolophus affinis*) | MF279192 |
| Morreton vesiculovirus (MORV) | *Vesiculovirus* | Colombia | 1986 | Sandfly (*Lutzomyia sp.*) | NC034508 |
| VS New Jersey vesiculovirus (VSNJV) | *Vesiculovirus* | Honduras | 1984 | Bovine | NC024473 |
| Chandipura vesiculovirus (CHNV) | *Vesiculovirus* | India | 2004 | Human | NC020805 |
| Isfahan vesiculovirus (ISFV) | *Vesiculovirus* | Iran | 1975 | Sandfly (*Phlebotomus papatasi*) | NC020806 |
| Radi vesiculovirus (RADIV) | *Vesiculovirus* | Italy | 1982 | Sandfly (*Phlebotomus perfiliewi*) | KM205024 |
| Perinet vesiculovirus (PERV) | *Vesiculovirus* | Madagascar | 2008 | Mosquito | NC025394 |
| Yug Bogdanovac vesiculovirus (YBV) | *Vesiculovirus* | Serbia | 2011 | Sandfly (Vero-E6 cells) | NC025378 |
| Cocal vesiculovirus (COCV) | *Vesiculovirus* | Trinidad | 1961 | Rodent | EU373657 |
| American bat vesiculovirus (ABV) | *Vesiculovirus* | USA | 2008 | Bat (*Eptesicus fuscus*) | NC022755 |
| Malpais Spring vesiculovirus (MSPV) | *Vesiculovirus* | USA | 1985 | Mosquito (*Aedes campestris*) | NC025364 |
| VS Indiana vesiculovirus (VSIV) | *Vesiculovirus* | USA | 1998 | Horse | NC001560 |
| Qiongzhong bat virus 1127 (QZBV_1127) | *Vesiculovirus* | China | 2007 | Bat (*Rhinolophus affinis*) | MN607593 |
| Yinshui bat virus 1017 (YSBV_1017) | *Vesiculovirus* | China | 2007 | Bat (*Rhinolophus sinica*) | MN607594 |
| Yinshui bat virus D170001 (YSBV_D170001) | *Vesiculovirus* | China | 2018 | Bat (*Rhinolophus sinica*) | MN607595 |
| Yinshui bat virus D170022 (YSBV_D170022) | *Vesiculovirus* | China | 2019 | Bat (*Rhinolophus sinica*) | MN607596 |
| Yinshui bat virus D170190 (YSBV_D170190) | *Vesiculovirus* | China | 2020 | Bat (*Rhinolophus sinica*) | MN607597 |
| Bughendera virus (BUGV) | *Ledantevirus* | Uganda | 2017 | Batfly (Dipseliopoda sp.) | MT325641 |
| Mediterranean bat virus (MBV_2012096) | *Vesiculovirus* | Spain | 2012 | Bat (*Miniopterus schreibersii*) | MW557328 (this studiy) |
| Mediterranean bat virus (MBV_A08011) | *Vesiculovirus* | Algeria | 2008 | Bat (*Rhinolophus ferrumequinum*) | MW557329 (this study) |
| Mediterranean bat virus (MBV_A08065) | *Vesiculovirus* | Algeria | 2008 | Bat (*Rhinolophus euryale*) | MW557330 (this study) |
| Mediterranean bat virus (MBV_A09061) | *Vesiculovirus* | Algeria | 2009 | Bat (*Rhinolophus ferrumequinum*) | MW557331 (this study) |
| Mediterranean bat virus (MBV_A09097) | *Vesiculovirus* | Algeria | 2009 | Bat (*Rhinolophus ferrumequinum*) | MW557332 (this study) |
| Mediterranean bat virus (MBV_A09145) | *Vesiculovirus* | Algeria | 2009 | Bat (*Rhinolophus ferrumequinum*) | MW557333 (this study) |
| Mediterranean bat virus (MBV_A09151) | *Vesiculovirus* | Algeria | 2009 | Bat (*Rhinolophus ferrumequinum*) | MW557334 (this study) |
| Mediterranean bat virus (MBV_A09153) | *Vesiculovirus* | Algeria | 2009 | Bat (*Rhinolophus ferrumequinum*) | MW557335 (this study) |
| Mediterranean bat virus (MBV_A09181) | *Vesiculovirus* | Algeria | 2009 | Bat (*Rhinolophus ferrumequinum*) | NC_076939 (this study) |
| Mediterranean bat virus (MBV_A09193) | *Vesiculovirus* | Algeria | 2009 | Bat (*Rhinolophus ferrumequinum*) | MW557337 (this study) |
| Mediterranean bat virus (MBV_A09197) | *Vesiculovirus* | Algeria | 2009 | Bat (*Rhinolophus ferrumequinum*) | MW557338 (this study) |
| Mediterranean bat virus (MBV_M08013) | *Vesiculovirus* | Morocco | 2008 | Bat (*Rhinolophus ferrumequinum*) | MW557339 (this study) |
| Mediterranean bat virus (MBV_M08017) | *Vesiculovirus* | Morocco | 2008 | Bat (*Rhinolophus ferrumequinum*) | MW557340 (this study) |
| Mediterranean bat virus (MBV_M08051) | *Vesiculovirus* | Morocco | 2008 | Bat (*Rhinolophus ferrumequinum*) | MW557341 (this study) |
| Mediterranean bat virus (MBV_M09005) | *Vesiculovirus* | Morocco | 2009 | Bat (*Rhinolophus ferrumequinum*) | MW557342 (this study) |
| Mediterranean bat virus (MBV_M09009) | *Vesiculovirus* | Morocco | 2009 | Bat (*Rhinolophus ferrumequinum*) | MW557343 (this study) |
| American bat vesiculovirus strain 20-0654 (ABV_20-0654) | *Vesiculovirus* | USA | 2020 | Bat (*Eptesicus fuscus*) | MT561344 |
| American bat vesiculovirus strain 20-7011 (ABV_20-7011) | *Vesiculovirus* | USA | 2020 | Bat (*Eptesicus fuscus*) | MT561345 |
| American bat vesiculovirus strain 20-7177 (ABV_20-7177) | *Vesiculovirus* | USA | 2020 | Bat (*Eptesicus fuscus*) | MT561346 |
| American bat vesiculovirus strain 20-8862 (ABV_20-8862) | *Vesiculovirus* | USA | 2020 | Bat (*Eptesicus fuscus*) | MT561347 |
