# Supplementary material for: Broad geographical circulation of a novel vesiculovirus in bats in the Mediterranean region
Source: PLoS Negl Trop Dis. 2025 Jun 12;19(6):e0013172. doi: 10.1371/journal.pntd.0013172 (PMC12193708; doi:10.1371/journal.pntd.0013172)
Supplement: S9 Table — (DOCX) [file pntd.0013172.s013.docx]

**Table S9:** Details and NGS results of the 23 bat samples positive for rhabdovirus detection by the pan-rhabdo RT-nqPCR.

| **Sample number** | **Sample type** | **Bat species** | **Sex** | **Country** | **Cave** | **Collection date** | **NGS result** |
| --- | --- | --- | --- | --- | --- | --- | --- |
| 2012086 | Oral swab | *Miniopterus schreibersii* | Male | Spain | I (A. Daví) | 10/12/2012 | Few reads |
| 2012088 | Oral swab | *Miniopterus schreibersii* | Female | Spain | I (A. Daví) | 10/12/2012 | Few reads |
| 2012094 | Oral swab | *Miniopterus schreibersii* | Female | Spain | I (A. Daví) | 10/12/2012 | Few reads |
| 2012096 | Oral swab | *Miniopterus schreibersii* | Male | Spain | I (A. Daví) | 10/12/2012 | Nearly full genome |
| 2012098 | Oral swab | *Miniopterus schreibersii* | Male | Spain | I (A. Daví) | 10/12/2012 | Few reads |
| 2012100 | Oral swab | *Miniopterus schreibersii* | Male | Spain | I (A. Daví) | 10/12/2012 | Few reads |
| M08013 | Blood | *Rhinolophus ferrumequinum* | Male | Morocco | II (Ghar-Knadel) | 25/06/2008 | Nearly full genome |
| M08017 | Blood | *Rhinolophus ferrumequinum* | Female | Morocco | II (Ghar-Knadel) | 25/06/2008 | Nearly full genome |
| M08051 | Blood | *Rhinolophus ferrumequinum* | Female | Morocco | II (Ghar-Knadel) | 25/06/2008 | Nearly full genome |
| M080113 | Blood | *Rhinolophus euryale* | Male | Morocco | III (Kef el Ghar) | 25/06/2008 | Few reads |
| M09005 | Blood | *Rhinolophus ferrumequinum* | Male | Morocco | IV (Ifri N' Caid) | 13/05/2009 | Nearly full genome |
| M09009 | Blood | *Rhinolophus ferrumequinum* | Male | Morocco | IV (Ifri N' Caid) | 13/05/2009 | Nearly full genome |
| A08011 | Blood | *Rhinolophus ferrumequinum* | Female | Algeria | V (Chrea) | 04/04/2008 | Nearly full genome |
| A09145 | Blood | *Rhinolophus ferrumequinum* | Male | Algeria | V (Chera) | 05/05/2009 | Nearly full genome |
| A09151 | Blood | *Rhinolophus ferrumequinum* | Male | Algeria | V (Chera) | 05/05/2009 | Nearly full genome |
| A09153 | Blood | *Rhinolophus ferrumequinum* | Male | Algeria | V (Chera) | 05/05/2009 | Nearly full genome |
| A09181 | Blood | *Rhinolophus ferrumequinum* | Male | Algeria | V (Chera) | 05/05/2009 | Nearly full genome |
| A09187 | Blood | *Rhinolophus ferrumequinum* | Female | Algeria | V (Chera) | 05/05/2009 | Few reads |
| A09193 | Blood | *Rhinolophus ferrumequinum* | Male | Algeria | V (Chera) | 05/05/2009 | Nearly full genome |
| A09197 | Blood | *Rhinolophus ferrumequinum* | Male | Algeria | V (Chera) | 05/05/2009 | Nearly full genome |
| A08065 | Blood | *Rhinolophus euryale* | Female | Algeria | VI (Aokas) | 06/04/2008 | Nearly full genome |
| A09061 | Blood | *Rhinolophus ferrumequinum* | Female | Algeria | VI (Aokas) | 02/05/2009 | Nearly full genome |
| A09097 | Blood | *Rhinolophus ferrumequinum* | Male | Algeria | VI (Aokas) | 03/05/2009 | Nearly full genome |
