# Supplementary material for: Broad geographical circulation of a novel vesiculovirus in bats in the Mediterranean region
Source: PLoS Negl Trop Dis. 2025 Jun 12;19(6):e0013172. doi: 10.1371/journal.pntd.0013172 (PMC12193708; doi:10.1371/journal.pntd.0013172)
Supplement: S10 Table — (DOCX) [file pntd.0013172.s014.docx]

**Table S10**: Pairwise nucleotide comparison of the 16 bat rhabdovirus genomes expressed in nucleotide identity (%), after ORF concatenation for each of them.

| **Pairwise nucleotide identity of concatenated ORFs (%)** | | | | | | | | | | | | | | | | |
| --- | --- | --- | --- | --- | --- | --- | --- | --- | --- | --- | --- | --- | --- | --- | --- | --- |
| **Isoale** | **A09181** | **A09197** | **A09145** | **A09193** | **A08065** | **A09097** | **A09061** | **A09151** | **A09153** | **A08011** | **M09005** | **M09009** | **2012096** | **M08017** | **M08051** | **M08013** |
| **A09181** | 100 |  |  |  |  |  |  |  |  |  |  |  |  |  |  |  |
| **A09197** | 99.9 | 100 |  |  |  |  |  |  |  |  |  |  |  |  |  |  |
| **A09145** | 99.9 | 99.9 | 100 |  |  |  |  |  |  |  |  |  |  |  |  |  |
| **A09193** | 99.9 | 99.9 | 99.9 | 100 |  |  |  |  |  |  |  |  |  |  |  |  |
| **A08065** | 99.2 | 99.2 | 99.2 | 99.2 | 100 |  |  |  |  |  |  |  |  |  |  |  |
| **A09097** | 99.8 | 99.8 | 99.8 | 99.8 | 99.4 | 100 |  |  |  |  |  |  |  |  |  |  |
| **A09061** | 99.7 | 99.7 | 99.7 | 99.7 | 99.3 | 99.9 | 100 |  |  |  |  |  |  |  |  |  |
| **A09151** | 99 | 99 | 99 | 99 | 98.4 | 99 | 99 | 100 |  |  |  |  |  |  |  |  |
| **A09153** | 99 | 99 | 99 | 99 | 98.4 | 99 | 99 | 99.9 | 100 |  |  |  |  |  |  |  |
| **A08011** | 98.6 | 98.6 | 98.6 | 98.6 | 98.8 | 98.7 | 98.6 | 99.5 | 99.5 | 100 |  |  |  |  |  |  |
| **M09005** | 97.3 | 97.3 | 97.3 | 97.3 | 97.8 | 97.3 | 97.3 | 97.5 | 97.5 | 97.9 | 100 |  |  |  |  |  |
| **M09009** | 97.9 | 97.9 | 97.9 | 97.9 | 97.4 | 98 | 97.9 | 98.1 | 98.1 | 97.8 | 99.3 | 100 |  |  |  |  |
| **2012096** | 97.8 | 97.8 | 97.8 | 97.9 | 97.3 | 97.9 | 97.8 | 98 | 98 | 97.6 | 99.2 | 99.8 | 100 |  |  |  |
| **M08017** | 97.5 | 97.5 | 97.5 | 97.5 | 97.9 | 97.5 | 97.5 | 97.6 | 97.6 | 98 | 99 | 98.7 | 98.5 | 100 |  |  |
| **M08051** | 97.9 | 97.9 | 97.9 | 98 | 97.4 | 98 | 97.9 | 98.1 | 98.1 | 97.7 | 98.5 | 99.1 | 99 | 99.5 | 100 |  |
| **M08013** | 97.9 | 97.9 | 97.9 | 98 | 97.4 | 98 | 97.9 | 98.1 | 98.1 | 97.7 | 98.5 | 99.1 | 99 | 99.5 | 99.9 | 100 |
