# Supplementary material for: Broad geographical circulation of a novel vesiculovirus in bats in the Mediterranean region
Source: PLoS Negl Trop Dis. 2025 Jun 12;19(6):e0013172. doi: 10.1371/journal.pntd.0013172 (PMC12193708; doi:10.1371/journal.pntd.0013172)
Supplement: S11 Table — Identities were calculated as pairwise deletion using MEGA7.0. (DOCX) [file pntd.0013172.s015.docx]

**Table S11.** Amino acid identities (%) of the P, G and L proteins between the 16 isolates of Mediterranean bat virus (MBV). Identities were calculated as pairwise deletion using MEGA7.0.

|  | **Pairwise amino acid identity (%)** | | | | | | | | | | | | | | | |
| --- | --- | --- | --- | --- | --- | --- | --- | --- | --- | --- | --- | --- | --- | --- | --- | --- |
|  | **2012096** | **M09005** | **M09009** | **M08013** | **M08017** | **M08051** | **A08011** | **A08065** | **A09061** | **A09097** | **A09145** | **A09151** | **A09153** | **A09181** | **A09193** | **A09197** |
| **P protein** |  |  |  |  |  |  |  |  |  |  |  |  |  |  |  |  |
| 2012096 | 100 |  |  |  |  |  |  |  |  |  |  |  |  |  |  |  |
| M09005 | 100 | 100 |  |  |  |  |  |  |  |  |  |  |  |  |  |  |
| M09009 | 100 | 100 | 100 |  |  |  |  |  |  |  |  |  |  |  |  |  |
| M08013 | 99.1 | 99.1 | 99.1 | 100 |  |  |  |  |  |  |  |  |  |  |  |  |
| M08017 | 99.1 | 99.1 | 99.1 | 100 | 100 |  |  |  |  |  |  |  |  |  |  |  |
| M08051 | 99.1 | 99.1 | 99.1 | 100 | 100 | 100 |  |  |  |  |  |  |  |  |  |  |
| A08011 | 98.6 | 98.6 | 98.6 | 98.6 | 98.6 | 98.6 | 100 |  |  |  |  |  |  |  |  |  |
| A08065 | 99.1 | 99.1 | 99.1 | 99.1 | 99.1 | 99.1 | 99.5 | 100 |  |  |  |  |  |  |  |  |
| A09061 | 99.1 | 99.1 | 99.1 | 99.1 | 99.1 | 99.1 | 99.5 | 100 | 100 |  |  |  |  |  |  |  |
| A09097 | 99.1 | 99.1 | 99.1 | 99.1 | 99.1 | 99.1 | 99.5 | 100 | 100 | 100 |  |  |  |  |  |  |
| A09145 | 99.1 | 99.1 | 99.1 | 99.1 | 99.1 | 99.1 | 99.5 | 100 | 100 | 100 | 100 |  |  |  |  |  |
| A09151 | 98.6 | 98.6 | 98.6 | 98.6 | 98.6 | 98.6 | 100 | 99.5 | 99.5 | 99.5 | 99.5 | 100 |  |  |  |  |
| A09153 | 98.6 | 98.6 | 98.6 | 98.6 | 98.6 | 98.6 | 100 | 99.5 | 99.5 | 99.5 | 99.5 | 100 | 100 |  |  |  |
| A09181 | 99.1 | 99.1 | 99.1 | 99.1 | 99.1 | 99.1 | 99.5 | 100 | 100 | 100 | 100 | 99.5 | 99.5 | 100 |  |  |
| A09193 | 99.1 | 99.1 | 99.1 | 99.1 | 99.1 | 99.1 | 99.5 | 100 | 100 | 100 | 100 | 99.5 | 99.5 | 100 | 100 |  |
| A09197 | 99.1 | 99.1 | 99.1 | 99.1 | 99.1 | 99.1 | 99.5 | 100 | 100 | 100 | 100 | 99.5 | 99.5 | 100 | 100 | 100 |
| **G protein** |  |  |  |  |  |  |  |  |  |  |  |  |  |  |  |  |
| 2012096 | 100 |  |  |  |  |  |  |  |  |  |  |  |  |  |  |  |
| M09005 | 99.6 | 100 |  |  |  |  |  |  |  |  |  |  |  |  |  |  |
| M09009 | 99.8 | 99.8 | 100 |  |  |  |  |  |  |  |  |  |  |  |  |  |
| M08013 | 99.4 | 99.4 | 99.6 | 100 |  |  |  |  |  |  |  |  |  |  |  |  |
| M08017 | 99.4 | 99.4 | 99.6 | 100 | 100 |  |  |  |  |  |  |  |  |  |  |  |
| M08051 | 99.4 | 99.4 | 99.6 | 100 | 100 | 100 |  |  |  |  |  |  |  |  |  |  |
| A08011 | 99 | 99 | 99.2 | 98.8 | 98.8 | 98.8 | 100 |  |  |  |  |  |  |  |  |  |
| A08065 | 98.8 | 98.8 | 99 | 98.6 | 98.6 | 98.6 | 99.8 | 100 |  |  |  |  |  |  |  |  |
| A09061 | 98.8 | 98.8 | 99 | 98.6 | 98.6 | 98.6 | 99.8 | 100 | 100 |  |  |  |  |  |  |  |
| A09097 | 98.8 | 98.8 | 99 | 98.6 | 98.6 | 98.6 | 99.8 | 100 | 100 | 100 |  |  |  |  |  |  |
| A09145 | 98.8 | 98.8 | 99 | 98.6 | 98.6 | 98.6 | 99.8 | 100 | 100 | 100 | 100 |  |  |  |  |  |
| A09151 | 99 | 99 | 99.2 | 98.8 | 98.8 | 98.8 | 100 | 99.8 | 99.8 | 99.8 | 99.8 | 100 |  |  |  |  |
| A09153 | 99 | 99 | 99.2 | 98.8 | 98.8 | 98.8 | 100 | 99.8 | 99.8 | 99.8 | 99.8 | 100 | 100 |  |  |  |
| A09181 | 98.8 | 98.8 | 99 | 98.6 | 98.6 | 98.6 | 99.8 | 100 | 100 | 100 | 100 | 99.8 | 99.8 | 100 |  |  |
| A09193 | 98.8 | 98.8 | 99 | 98.6 | 98.6 | 98.6 | 99.8 | 100 | 100 | 100 | 100 | 99.8 | 99.8 | 100 | 100 |  |
| A09197 | 98.8 | 98.8 | 99 | 98.6 | 98.6 | 98.6 | 99.8 | 100 | 100 | 100 | 100 | 99.8 | 99.8 | 100 | 100 | 100 |
| **L protein** |  |  |  |  |  |  |  |  |  |  |  |  |  |  |  |  |
| 2012096 | 100 |  |  |  |  |  |  |  |  |  |  |  |  |  |  |  |
| M09005 | 99.7 | 100 |  |  |  |  |  |  |  |  |  |  |  |  |  |  |
| M09009 | 99.8 | 99.9 | 100 |  |  |  |  |  |  |  |  |  |  |  |  |  |
| M08013 | 99.7 | 99.8 | 99.9 | 100 |  |  |  |  |  |  |  |  |  |  |  |  |
| M08017 | 99.7 | 99.8 | 99.9 | 100 | 100 |  |  |  |  |  |  |  |  |  |  |  |
| M08051 | 99.7 | 99.8 | 99.9 | 100 | 100 | 100 |  |  |  |  |  |  |  |  |  |  |
| A08011 | 99 | 99.1 | 99.2 | 99.2 | 99.2 | 99.2 | 100 |  |  |  |  |  |  |  |  |  |
| A08065 | 99.1 | 99.2 | 99.3 | 99.3 | 99.3 | 99.3 | 99.5 | 100 |  |  |  |  |  |  |  |  |
| A09061 | 99.1 | 99.2 | 99.3 | 99.4 | 99.4 | 99.4 | 99.5 | 99.9 | 100 |  |  |  |  |  |  |  |
| A09097 | 99.1 | 99.2 | 99.3 | 99.4 | 99.4 | 99.4 | 99.5 | 99.9 | 100 | 100 |  |  |  |  |  |  |
| A09145 | 99.1 | 99.2 | 99.3 | 99.3 | 99.3 | 99.3 | 99.5 | 99.9 | 99.9 | 99.9 | 100 |  |  |  |  |  |
| A09151 | 99 | 99 | 99.1 | 99.2 | 99.2 | 99.2 | 99.8 | 99.5 | 99.6 | 99.6 | 99.5 | 100 |  |  |  |  |
| A09153 | 99 | 99 | 99.1 | 99.2 | 99.2 | 99.2 | 99.8 | 99.5 | 99.6 | 99.6 | 99.5 | 100 | 100 |  |  |  |
| A09181 | 99.1 | 99.2 | 99.3 | 99.3 | 99.3 | 99.3 | 99.5 | 99.9 | 99.9 | 99.9 | 100 | 99.5 | 99.5 | 100 |  |  |
| A09193 | 99.1 | 99.2 | 99.3 | 99.3 | 99.3 | 99.3 | 99.5 | 99.9 | 99.9 | 99.9 | 100 | 99.5 | 99.5 | 100 | 100 |  |
| A09197 | 99 | 99.1 | 99.2 | 99.3 | 99.3 | 99.3 | 99.4 | 99.8 | 99.9 | 99.9 | 99.9 | 99.5 | 99.5 | 99.9 | 99.9 | 100 |
