# Supplementary material for: Broad geographical circulation of a novel vesiculovirus in bats in the Mediterranean region
Source: PLoS Negl Trop Dis. 2025 Jun 12;19(6):e0013172. doi: 10.1371/journal.pntd.0013172 (PMC12193708; doi:10.1371/journal.pntd.0013172)
Supplement: S12 Table — Identities were calculated as pairwise deletion using MEGA7.0. (DOCX) [file pntd.0013172.s016.docx]

**Table S12.** Amino acid identities (%) of the 5 proteins (N, P, M, G and L) of the Mediterranean bat virus isolate 2012096 compared to other species among the genus *Vesiculovirus*. Identities were calculated as pairwise deletion using MEGA7.0.

|  | **Pairwise amino acid identity (%)** | | | | | | | | | | | | | | | | | | | |
| --- | --- | --- | --- | --- | --- | --- | --- | --- | --- | --- | --- | --- | --- | --- | --- | --- | --- | --- | --- | --- |
|  | **MBV (2012096)** | **YSBV (1017)** | **QZBV (1127)** | **JHBV** | **ABV** | **YBV** | **CARV** | **VSIV** | **VSAV** | **COCV** | **MORV** | **RADIV** | **MSPV** | **VSNJV** | **MARAV** | **JURV** | **PIRV** | **ISFV** | **CHNV** | **PERV** |
| **N Protein** |  |  |  |  |  |  |  |  |  |  |  |  |  |  |  |  |  |  |  |  |
| MBV_2012096 | 100 |  |  |  |  |  |  |  |  |  |  |  |  |  |  |  |  |  |  |  |
| YSBV_1017 | 75.5 | 100 |  |  |  |  |  |  |  |  |  |  |  |  |  |  |  |  |  |  |
| QZBV_1127 | 74.6 | 89.8 | 100 |  |  |  |  |  |  |  |  |  |  |  |  |  |  |  |  |  |
| JHBV | 74.6 | 89.5 | 99.7 | 100 |  |  |  |  |  |  |  |  |  |  |  |  |  |  |  |  |
| ABV | 53.5 | 54.2 | 54.7 | 54.4 | 100 |  |  |  |  |  |  |  |  |  |  |  |  |  |  |  |
| YBV | 47.8 | 46.7 | 47.4 | 47.4 | 48.5 | 100 |  |  |  |  |  |  |  |  |  |  |  |  |  |  |
| CARV | 46.9 | 45.5 | 45.5 | 45.5 | 46.4 | 53.3 | 100 |  |  |  |  |  |  |  |  |  |  |  |  |  |
| VSIV | 46.4 | 46.9 | 46.9 | 46.7 | 45.5 | 52.9 | 75.8 | 100 |  |  |  |  |  |  |  |  |  |  |  |  |
| VSAV | 46.2 | 45.5 | 45.5 | 45.3 | 44.6 | 53.3 | 74.8 | 84.8 | 100 |  |  |  |  |  |  |  |  |  |  |  |
| COCV | 46 | 45.5 | 45.7 | 45.5 | 45.3 | 51.7 | 73.9 | 83.6 | 85.3 | 100 |  |  |  |  |  |  |  |  |  |  |
| MORV | 46 | 47.1 | 45.5 | 45.5 | 46.2 | 52.2 | 74.8 | 90.5 | 83.4 | 83.4 | 100 |  |  |  |  |  |  |  |  |  |
| RADIV | 46 | 45.5 | 43.2 | 43.4 | 47.4 | 74.5 | 53.1 | 54.7 | 53.8 | 51.7 | 53.6 | 100 |  |  |  |  |  |  |  |  |
| MSPV | 45.5 | 46.9 | 45.8 | 45.8 | 46.5 | 51.9 | 55.2 | 55.2 | 54.3 | 55 | 53.8 | 54.2 | 100 |  |  |  |  |  |  |  |
| VSNJV | 45.5 | 47.4 | 46.2 | 46.2 | 46.9 | 52.9 | 72.5 | 69.1 | 69.4 | 70.1 | 68.7 | 54.5 | 55.5 | 100 |  |  |  |  |  |  |
| MARAV | 45.3 | 45 | 45 | 44.8 | 45.7 | 52.2 | 73.4 | 90 | 82.4 | 86.7 | 88.8 | 53.3 | 55.7 | 69.6 | 100 |  |  |  |  |  |
| JURV | 44.1 | 46.2 | 45.7 | 45.7 | 48.5 | 54 | 52.4 | 54.1 | 54.8 | 52.4 | 54.3 | 55.3 | 70.3 | 54.1 | 53.4 | 100 |  |  |  |  |
| PIRV | 43.7 | 46 | 43.9 | 43.9 | 48.6 | 51.2 | 49.5 | 52.5 | 51.4 | 50.9 | 52.1 | 53.9 | 61.5 | 51.4 | 51.6 | 62 | 100 |  |  |  |
| ISFV | 43.3 | 45.9 | 43.7 | 43.7 | 47.7 | 53.6 | 51.1 | 51.8 | 52.1 | 51.1 | 51.8 | 54.3 | 65.2 | 51.8 | 51.1 | 66.2 | 60.9 | 100 |  |  |
| CHNV | 42.8 | 42.8 | 42.6 | 42.6 | 47 | 48.9 | 50.2 | 50.4 | 48.1 | 48.1 | 50.4 | 50.9 | 58.1 | 50.2 | 50 | 61.4 | 55 | 58.3 | 100 |  |
| PERV | 38.3 | 38 | 36.6 | 36.6 | 39.6 | 42.5 | 45 | 45.3 | 45 | 45.3 | 45.3 | 44 | 52.8 | 46.2 | 45.7 | 49.8 | 51.6 | 47 | 43.7 | 100 |
| **P protein** |  |  |  |  |  |  |  |  |  |  |  |  |  |  |  |  |  |  |  |  |
| MBV_2012096 | 100 |  |  |  |  |  |  |  |  |  |  |  |  |  |  |  |  |  |  |  |
| YSBV_1017 | 21.1 | 100 |  |  |  |  |  |  |  |  |  |  |  |  |  |  |  |  |  |  |
| QZBV_1127 | 26.9 | 46 | 100 |  |  |  |  |  |  |  |  |  |  |  |  |  |  |  |  |  |
| JHBV | 27.8 | 48.8 | 86.6 | 100 |  |  |  |  |  |  |  |  |  |  |  |  |  |  |  |  |
| ABV | 11.6 | 9.7 | 9.4 | 9.8 | 100 |  |  |  |  |  |  |  |  |  |  |  |  |  |  |  |
| YBV | 12.3 | 14.8 | 13.6 | 12.9 | 9.4 | 100 |  |  |  |  |  |  |  |  |  |  |  |  |  |  |
| CARV | 12.7 | 9.5 | 10.7 | 11.7 | 14.6 | 11.9 | 100 |  |  |  |  |  |  |  |  |  |  |  |  |  |
| VSIV | 12.9 | 8.6 | 11.2 | 11.2 | 14.5 | 16.1 | 46.5 | 100 |  |  |  |  |  |  |  |  |  |  |  |  |
| VSAV | 10.1 | 8.1 | 11 | 11.3 | 15 | 15.8 | 44.4 | 53.5 | 100 |  |  |  |  |  |  |  |  |  |  |  |
| COCV | 10.4 | 10.4 | 10.9 | 11.3 | 17.5 | 16.4 | 44.6 | 58.2 | 60.2 | 100 |  |  |  |  |  |  |  |  |  |  |
| MORV | 13.5 | 8.9 | 11.8 | 11.8 | 16.2 | 18.8 | 45.4 | 67.7 | 53.7 | 61.8 | 100 |  |  |  |  |  |  |  |  |  |
| RADIV | 12.8 | 9.8 | 11.8 | 10 | 11 | 36.3 | 15.1 | 15.3 | 14.7 | 16.4 | 14.5 | 100 |  |  |  |  |  |  |  |  |
| MSPV | 10.3 | 11 | 10.1 | 10.1 | 15.6 | 12.4 | 16.4 | 17.4 | 16.4 | 18.1 | 17.6 | 15 | 100 |  |  |  |  |  |  |  |
| VSNJV | 10.1 | 8.4 | 8.8 | 9.8 | 14 | 11.2 | 37.8 | 29.8 | 32.4 | 33.2 | 30.8 | 12.8 | 15.1 | 100 |  |  |  |  |  |  |
| MARAV | 12.6 | 9.7 | 10.9 | 10.9 | 16 | 18.1 | 46.8 | 62.1 | 58.2 | 62 | 64.4 | 15.3 | 15.7 | 33 | 100 |  |  |  |  |  |
| JURV | 8.9 | 8.7 | 10 | 10.6 | 14.1 | 13.6 | 17.1 | 14.6 | 13 | 14.9 | 13.9 | 14 | 20.8 | 17.4 | 13.3 | 100 |  |  |  |  |
| PIRV | 11.4 | 9.6 | 10.2 | 11.1 | 14.1 | 12.5 | 22.3 | 16.3 | 13.8 | 14.5 | 16 | 11.3 | 19.2 | 17.5 | 17.2 | 30.2 | 100 |  |  |  |
| ISFV | 7.3 | 7.7 | 10.4 | 10.7 | 13.6 | 13.6 | 15.5 | 14.9 | 15.9 | 14.9 | 13.9 | 11.2 | 17.5 | 15.5 | 13.6 | 28.7 | 26.7 | 100 |  |  |
| CHNV | 11 | 9.7 | 9.8 | 9.1 | 14.5 | 13.9 | 18.4 | 18.3 | 19.1 | 19.1 | 16.2 | 13.5 | 22.8 | 14.4 | 16.4 | 22.9 | 26.6 | 23.8 | 100 |  |
| PERV | 8.2 | 7.3 | 12.4 | 10.4 | 14.6 | 11.2 | 15.5 | 15.8 | 17.2 | 16.5 | 15.8 | 13.7 | 22.9 | 18.8 | 14.9 | 31.2 | 26.6 | 27.6 | 25 | 100 |
| **M protein** |  |  |  |  |  |  |  |  |  |  |  |  |  |  |  |  |  |  |  |  |
| MBV_2012096 | 100 |  |  |  |  |  |  |  |  |  |  |  |  |  |  |  |  |  |  |  |
| YSBV_1017 | 61.5 | 100 |  |  |  |  |  |  |  |  |  |  |  |  |  |  |  |  |  |  |
| QZBV_1127 | 56.7 | 73.5 | 100 |  |  |  |  |  |  |  |  |  |  |  |  |  |  |  |  |  |
| JHBV | 56.2 | 74.5 | 95.1 | 100 |  |  |  |  |  |  |  |  |  |  |  |  |  |  |  |  |
| ABV | 40.4 | 40.9 | 39.5 | 40 | 100 |  |  |  |  |  |  |  |  |  |  |  |  |  |  |  |
| YBV | 18.9 | 19.7 | 19.3 | 20.1 | 23.8 | 100 |  |  |  |  |  |  |  |  |  |  |  |  |  |  |
| CARV | 19.2 | 18.8 | 18.4 | 17.9 | 19.2 | 17.7 | 100 |  |  |  |  |  |  |  |  |  |  |  |  |  |
| VSIV | 18.7 | 17.9 | 18.7 | 19.6 | 19.2 | 20 | 55.6 | 100 |  |  |  |  |  |  |  |  |  |  |  |  |
| VSAV | 18.3 | 17.9 | 19.2 | 19.6 | 22.7 | 21.2 | 55.6 | 74.6 | 100 |  |  |  |  |  |  |  |  |  |  |  |
| COCV | 19.6 | 18.3 | 20 | 20.5 | 21.3 | 18.8 | 57.3 | 75.1 | 81.2 | 100 |  |  |  |  |  |  |  |  |  |  |
| MORV | 18.3 | 17.4 | 17.9 | 18.3 | 20 | 19.2 | 60 | 82.9 | 77.2 | 79 | 100 |  |  |  |  |  |  |  |  |  |
| RADIV | 20.9 | 19.2 | 19.2 | 19.6 | 26.3 | 54.1 | 20.3 | 19.5 | 21.5 | 20.3 | 21.1 | 100 |  |  |  |  |  |  |  |  |
| MSPV | 22 | 22.8 | 23.2 | 24.1 | 25.8 | 27.7 | 23.8 | 27.2 | 26.8 | 25.9 | 26.3 | 29.4 | 100 |  |  |  |  |  |  |  |
| VSNJV | 18.3 | 15.2 | 17.9 | 18.7 | 18.7 | 20 | 54.5 | 62.1 | 60.8 | 60.8 | 62.6 | 20.2 | 24.5 | 100 |  |  |  |  |  |  |
| MARAV | 18.3 | 17 | 19.2 | 20 | 20.5 | 20 | 56.9 | 79.9 | 77.2 | 79.9 | 82.9 | 22.3 | 26.3 | 63.9 | 100 |  |  |  |  |  |
| JURV | 21.9 | 21.3 | 20.5 | 20.5 | 27 | 29.5 | 27.4 | 26.6 | 26.6 | 26.1 | 28.3 | 28.3 | 41.6 | 29.4 | 28.3 | 100 |  |  |  |  |
| PIRV | 21 | 23.4 | 20.8 | 21.2 | 29.7 | 30.7 | 21.3 | 25.9 | 26.7 | 26.3 | 26.3 | 29.1 | 39.9 | 24.5 | 25.5 | 46.5 | 100 |  |  |  |
| ISFV | 22.1 | 20.7 | 22 | 22.9 | 28.1 | 32.3 | 23.8 | 26.3 | 26.8 | 27.2 | 25.9 | 32.9 | 43.8 | 27.1 | 27.2 | 53.7 | 47.4 | 100 |  |  |
| CHNV | 20.7 | 20.6 | 21.1 | 20.6 | 27.5 | 29.5 | 23.7 | 25 | 26.2 | 24.5 | 27.1 | 31.2 | 36.5 | 23.6 | 26.2 | 47.5 | 41.8 | 50.2 | 100 |  |
| PERV | 17.9 | 19 | 19.9 | 19.9 | 22.8 | 27.8 | 23.2 | 23.2 | 26.1 | 23.6 | 24.4 | 27.3 | 38.3 | 26 | 26.1 | 46 | 48.3 | 46.8 | 40.1 | 100 |
| **G protein** |  |  |  |  |  |  |  |  |  |  |  |  |  |  |  |  |  |  |  |  |
| MBV_2012096 | 100 |  |  |  |  |  |  |  |  |  |  |  |  |  |  |  |  |  |  |  |
| YSBV_1017 | 62.4 | 100 |  |  |  |  |  |  |  |  |  |  |  |  |  |  |  |  |  |  |
| QZBV_1127 | 59.3 | 71.9 | 100 |  |  |  |  |  |  |  |  |  |  |  |  |  |  |  |  |  |
| JHBV | 59.5 | 71.7 | 84.8 | 100 |  |  |  |  |  |  |  |  |  |  |  |  |  |  |  |  |
| ABV | 31.2 | 29.6 | 30.9 | 30.9 | 100 |  |  |  |  |  |  |  |  |  |  |  |  |  |  |  |
| YBV | 24.2 | 24.3 | 24.1 | 24.3 | 23.9 | 100 |  |  |  |  |  |  |  |  |  |  |  |  |  |  |
| CARV | 25.9 | 27.5 | 28.3 | 28.7 | 26.5 | 35.2 | 100 |  |  |  |  |  |  |  |  |  |  |  |  |  |
| VSIV | 27.5 | 29 | 28.6 | 28.6 | 23.4 | 34.1 | 54.1 | 100 |  |  |  |  |  |  |  |  |  |  |  |  |
| VSAV | 27.5 | 27.5 | 28.4 | 28.2 | 22.2 | 33.5 | 53.7 | 62.3 | 100 |  |  |  |  |  |  |  |  |  |  |  |
| COCV | 26.9 | 28 | 28.7 | 28.5 | 22.6 | 32.6 | 54.1 | 71.2 | 66.9 | 100 |  |  |  |  |  |  |  |  |  |  |
| MORV | 27.6 | 27.8 | 27.6 | 28 | 24.1 | 35.1 | 55.6 | 84.4 | 63.9 | 71.5 | 100 |  |  |  |  |  |  |  |  |  |
| RADIV | 24.9 | 24.9 | 24.2 | 24 | 23.9 | 67 | 34.9 | 34.3 | 33.8 | 34.1 | 35.5 | 100 |  |  |  |  |  |  |  |  |
| MSPV | 26.7 | 27.4 | 25.3 | 25.5 | 26.1 | 40.2 | 36.9 | 35.6 | 37.1 | 36.3 | 37 | 38.1 | 100 |  |  |  |  |  |  |  |
| VSNJV | 26.4 | 27.5 | 27.5 | 27.3 | 23.8 | 31.4 | 50.5 | 49.2 | 47.9 | 47.2 | 48.8 | 32 | 36.9 | 100 |  |  |  |  |  |  |
| MARAV | 27.7 | 28.1 | 28.6 | 28.4 | 23.4 | 33.7 | 54.6 | 77.3 | 63.9 | 73.8 | 77.4 | 35.2 | 36.1 | 49.7 | 100 |  |  |  |  |  |
| JURV | 26.3 | 27.8 | 27 | 26.4 | 25.2 | 41.8 | 38.5 | 36.4 | 36.8 | 38.7 | 37.3 | 40.1 | 48.6 | 36.7 | 37.1 | 100 |  |  |  |  |
| PIRV | 27 | 27.5 | 26 | 25.6 | 26.1 | 44.2 | 40.5 | 37.6 | 36.8 | 36.8 | 37.4 | 42.9 | 47.7 | 37 | 38.2 | 48.8 | 100 |  |  |  |
| ISFV | 27 | 27.9 | 26.4 | 26.4 | 24.4 | 40.1 | 38.4 | 38.4 | 36 | 40 | 39.6 | 39.2 | 47.9 | 37.1 | 37.5 | 53.5 | 50 | 100 |  |  |
| CHNV | 27.4 | 26.7 | 26.1 | 26.1 | 23.7 | 41.9 | 41.1 | 38.3 | 39.2 | 39.6 | 39.8 | 41.5 | 48.3 | 37 | 39.8 | 52.4 | 51.5 | 54.1 | 100 |  |
| PERV | 27.7 | 28 | 26.5 | 26.7 | 24.8 | 43.3 | 38.7 | 37.7 | 38.9 | 37.3 | 38.2 | 41.6 | 49 | 38.2 | 37.9 | 51 | 56.5 | 49.1 | 50.9 | 100 |
| **L protein** |  |  |  |  |  |  |  |  |  |  |  |  |  |  |  |  |  |  |  |  |
| MBV_2012096 | 100 |  |  |  |  |  |  |  |  |  |  |  |  |  |  |  |  |  |  |  |
| YSBV_1017 | 67 | 100 |  |  |  |  |  |  |  |  |  |  |  |  |  |  |  |  |  |  |
| QZBV_1127 | 66 | 75.5 | 100 |  |  |  |  |  |  |  |  |  |  |  |  |  |  |  |  |  |
| JHBV | 65.9 | 76 | 91.3 | 100 |  |  |  |  |  |  |  |  |  |  |  |  |  |  |  |  |
| ABV | 56.8 | 57.5 | 57.6 | 57 | 100 |  |  |  |  |  |  |  |  |  |  |  |  |  |  |  |
| YBV | 53.9 | 53.2 | 52.7 | 53 | 54.8 | 100 |  |  |  |  |  |  |  |  |  |  |  |  |  |  |
| CARV | 53.1 | 53 | 52.8 | 52.5 | 54 | 57.8 | 100 |  |  |  |  |  |  |  |  |  |  |  |  |  |
| VSIV | 52.5 | 51.9 | 52.2 | 52.1 | 53.4 | 56.7 | 69.7 | 100 |  |  |  |  |  |  |  |  |  |  |  |  |
| VSAV | 53.5 | 53.2 | 52.4 | 52.1 | 54 | 57.4 | 69.7 | 75.5 | 100 |  |  |  |  |  |  |  |  |  |  |  |
| COCV | 52.6 | 53.3 | 52.5 | 52.5 | 53.2 | 57.7 | 68.9 | 76.4 | 78.2 | 100 |  |  |  |  |  |  |  |  |  |  |
| MORV | 52.5 | 52.8 | 53.3 | 52.8 | 53.1 | 57.3 | 69 | 80.3 | 75.3 | 77.2 | 100 |  |  |  |  |  |  |  |  |  |
| RADIV | 54.7 | 54.5 | 53.5 | 53.2 | 55.9 | 72.3 | 57.5 | 56.8 | 57.4 | 57.8 | 56.7 | 100 |  |  |  |  |  |  |  |  |
| MSPV | 53.9 | 54.2 | 55.1 | 54.5 | 56.7 | 59.3 | 59.2 | 58.9 | 59 | 59.1 | 58.4 | 61 | 100 |  |  |  |  |  |  |  |
| VSNJV | 52.5 | 52.4 | 52.4 | 52.2 | 53.8 | 56.6 | 69.7 | 65.8 | 67.3 | 65.6 | 65.9 | 57.4 | 57.9 | 100 |  |  |  |  |  |  |
| MARAV | 52.6 | 52.3 | 52 | 51.9 | 52.6 | 57.2 | 68.5 | 78 | 77.1 | 78.9 | 78.7 | 56.8 | 58.4 | 65.7 | 100 |  |  |  |  |  |
| JURV | 52.9 | 53.4 | 53.2 | 53.2 | 56.9 | 58.1 | 58.4 | 58.1 | 57.9 | 57.9 | 57.2 | 60.1 | 67.4 | 57.5 | 57.6 | 100 |  |  |  |  |
| PIRV | 53 | 52.8 | 53.7 | 53.2 | 56.9 | 59.2 | 58.7 | 57.3 | 57.6 | 57 | 57.3 | 60.1 | 67.6 | 57.9 | 56.9 | 66.5 | 100 |  |  |  |
| ISFV | 52.9 | 54.8 | 53.9 | 53.9 | 55.6 | 58.6 | 58.8 | 58.2 | 58.3 | 58.4 | 58.3 | 61 | 68.6 | 57.5 | 58.5 | 66.1 | 66.4 | 100 |  |  |
| CHNV | 53.3 | 54.3 | 54.5 | 54 | 55.3 | 59.8 | 58.6 | 58.8 | 58.2 | 57.8 | 58 | 61.6 | 67.4 | 57.6 | 58.5 | 66.5 | 66.7 | 68.5 | 100 |  |
| PERV | 52.4 | 53.7 | 53.5 | 53.5 | 54.8 | 58.3 | 58.7 | 57.1 | 57.3 | 57.8 | 58.1 | 59.6 | 66.7 | 57.1 | 57.3 | 64.5 | 66.9 | 66.5 | 66.3 | 100 |
