# Supplementary material for: Broad geographical circulation of a novel vesiculovirus in bats in the Mediterranean region
Source: PLoS Negl Trop Dis. 2025 Jun 12;19(6):e0013172. doi: 10.1371/journal.pntd.0013172 (PMC12193708; doi:10.1371/journal.pntd.0013172)
Supplement: Attachment — Submitted filename: Rebuttal_letter_manuscript_Luo_al_PlosNTD_MBV_R1_210325.docx [file pntd.0013172.s017.docx]

**Ref: PNTD-D-24-01255**

**Broad geographical circulation of a novel vesiculovirus in bats in the Mediterranean region**

**Specific answers to the reviewers' comments: (text in blue)** (text from the Reviewers is indicated in italic):

Methods

*Reviewer #1: (No Response)*

*Reviewer #2: We do not feel that any new analyses are needed. However, there are a number of methodological and experimental details that could be clarified and condensed. The molecular methods (e.g. cDNA synthesis) are admirably thorough, but a lot of the standard methodology can be moved to the supplement and authors can highlight where their protocol deviated from the manufacturer's instructions. We appreciated them sharing these details and they will no doubt be useful to some but for the sake of the flow of the paper and people who are not directly replicating their experiments, it would be better in the supplement.*

We would like to thank The Reviewer 2 for these positive and constructive comments. As suggested, we have moved some of the ‘basic’ or detailed methodological aspects as additional data, as illustrated for Pan-rhabdovirus PCR method validation section or the Virus genome sequencing section.

*152-154: subordinate clause is missing a verb.*

We replaced “with” with “including”.

*252: should be “were” instead of “was”.*

We replaced “was” with “were”.

*251-253 is basically identical to 261-263.*

We deleted this repetition.

*Section 2.1 and 2.2: were all samples preserved and stored in the same way prior to their shipment to Institut Pasteur? This could seriously impact sample degradation.*

We would like to thank Reviewer 2 for this relevant remark. We added the following information: “After collection in the field, the quality of the samples was maintained as well as possible until their transfer to the laboratories of the countries concerned, in particular through the use of a preservation medium (RNAlater) for the oral swabs, and refrigerated coolers for the blood samples. Depending on the geographical area, the brain was obtained directly in the laboratory after transferring of the refrigerated cadaver and dissection, or in the field after dissection and use of a preservation medium (RNAlater).”

*Section 2.3-2.5: positive and negative controls for virus are included, but was there any confirmation of successful RNA extraction/cDNA synthesis? This is important to determine whether negatives are true negatives or lack of starting material.*

Again, we would like to thank Reviewer 2 for this important remark. Due to time and cost constraints, it was not possible to carry out an individual cDNA extraction/control for all samples, particularly through a specific PCR targeting an endogenous gene (b-actin or GAPDH), or by spiking exogenous RNA. However, negative controls were included in each extraction series, as well as positive and negative controls during the cDNA step. Finally, for most of the samples, estimation of the extracted nucleic acids was carried out by spectrophotometric assay (Nanodrop device). We also added some information in the revised version of the manuscript: “Negative controls were included in each extraction series, as well as positive and negative controls during the cDNA step. For most of the samples, estimation of the extracted nucleic acids was carried out by spectrophotometric assay (NanoDrop 2000, Thermo Scientific).”.

*3. Geographic Focus of Samples:*

*The manuscript focuses on samples from Europe and Africa, but the rationale for this geographic selection is not clear. Why were these particular samples chosen? Is there a known difference in rhabdovirus prevalence or diversity in these regions that makes them particularly relevant to the study? A more thorough explanation of the importance of these geographic regions in the context of viral emergence and zoonotic transmission would be helpful.*

As suggested by Reviewer 2, we have added information in the introduction section on the choice of Europe and Africa as target regions for this study, as follow: “This technique was then applied to analyze a large collection of bat samples from Africa and Europe, two different geographical regions of particular interest for rhabdoviruses, in a context of viral emergence and zoonotic transmission. For example, of the 22 different lyssaviruses discovered to date in the world, 6 have been identified in Europe and 6 others in Africa (https://ictv.global/taxonomy), whose transmission to humans or other mammals has been demonstrated (Dacheux et al., 2009; Regnault et al., 2021). In addition, other bat-related rhabdoviruses have also recently been discovered in these geographical regions, such as two new members of Lendatevirus genus with Vaprio virus in Pipistrellus pipistrellus bat species in Italy (Lelli et al., 2018), or Kanyawara virus in pteropodid bats in Uganda (Goldberg et al., 2017).”

Results

*Reviewer #1: (No Response)*

*Reviewer #2: They did a great job testing their assay and the results seem robust though we would like clarity on whether sample degradation might have impacted their detection results. Other comments below:*

We would like to thank Reviewer 2 for this positive comment. The question of the impact of the degradation of the samples on the detection results is crucial and does indeed need to be clarified.

As indicated in response to the similar comment made in the Method part, we indicated in the revised version of the manuscript: “After collection in the field, the quality of the samples was maintained as well as possible until their transfer to the laboratories of the countries concerned, in particular through the use of a preservation medium (RNAlater) for the saliva swabs, or refrigerated coolers for the blood samples. Depending on the geographical area, the brain was obtained directly in the laboratory after transfer of the refrigerated cadaver and dissection, or in the field after dissection and use of a preservation medium (RNAlater).”.

We also added, in the Discussion section, the following information: “Although high, we cannot completely exclude a potential negative impact of the quality of some samples on the results of this prevalence, despite the implementation of measures aimed at maximizing the conservation of samples taken in the field (quality of sampling, use of a conservation medium such as RNAlater or TRIzol, conditions of transport and refrigerated storage, etc.).”.

*Line 300: It’s an interesting finding that all brains were negative, but they aren’t discussed after this point.*

Following the comment from Reviewer 2, we added more information in the Discussion section on analyses related to brain tissue, as follow: “In our study, the brain was the only organ analyzed, and all samples were found negative for rhabdovirus detection. This result was not surprising because the prevalence found in other similar studies remains low, particularly in individuals appearing asymptomatic (e.g. 0.6% in China, ref Luo 2021). In addition, we deliberately excluded bat individuals that had previously been confirmed as rabid (particularly those from the biocollection originating in France), which further reduced the likelihood of finding positive samples. Regarding the detection results of MBV, saliva, blood, and brain samples were not collected from the same bat individuals and were gathered across different countries and years. For example, only 16 brain samples were collected from Morocco from 3 different bat species, which limits the interpretation of the results regarding the tropism of this virus for the brain.”.

*Despite the assay being pan-rhabdovirus there were no detections of lyssaviruses even though some samples were originally collected for the purpose of lyssavirus monitoring. Were bats with EBLV or other lyssaviruses intentionally excluded? If not, is the lack of finding of EBLV surprising?*

As indicated previously, we deliberately excluded bat individuals that had previously been confirmed as rabid (particularly those from the biocollection originating in France), which further reduced the likelihood of finding positive samples. Indeed, after validating our technique (using samples of bats naturally infected with EBLV-1, for example), we chose to focus only on individuals tested negative among those collected as part of lyssavirus surveillance, in order to potentially identify new rhabdoviruses. Furthermore, the prevalence of lyssaviruses or rhabdoviruses remains very low in bats, especially when they are asymptomatic.

*Figure 3: the labels of the grey ones especially the dark grey get hard to see; can you pull the text in front? Also it’s a little hard to tell what the boostrap values refer to in the R. affinis and R. sinicus clades.*

Modified accordingly.

*372-378 and figure S3: If in the figure the authors could denote where each sequence is from it would be easier for the reader. Also are the mutations listed here by geography referring to how many sites with mutations there were in these areas?*

We modified accordingly the Figure S3, with the addition of the location for each sequence. We also replaced “total” with “the overall number of mutations, regardless of geographical location…” to make it clear that this is the total number of transfers, regardless of geographical location. This distinction is indicated in the rest of the paragraph, in the manuscript.

Conclusions

*Reviewer #1: (No Response)*

*Reviewer #2: In general the conclusions are supported. We think the authors could improve the manuscript by streamlining it and removing interesting but irrelevant information (e.g. shape of rhabdoviruses) and focusing on the main applicability of their studies to infectious disease and public health.*

As suggested by Reviewer 2, we have removed some irrelevant information, such as “They are typically enveloped virions with bullet-shaped or bacilliform morphology, ranging in length from 100 to 460 nm and diameter from 45 to 100 nm.” in the Introduction section, and we have moved some information from the Materials and Methods section to the Supplementary Materials section. We also deleted some parts in the Discussion section.

*In particular, we highlight a few areas and specific lines:*

*Missing Emphasis on Importance: The broader significance of the study, especially in relation to viral emergence, zoonotic transmission, and the role of bats in rhabdovirus ecology, is not adequately highlighted. In particular, the importance of establishing a pan-animal rhabdovirus detection system should be emphasized more clearly. This could help in monitoring viral emergence and preventing zoonotic spillover, especially given the increasing frequency of novel virus discovery in bat populations.*

*Importance of Bats: The role of bats as key hosts for rhabdoviruses is mentioned in the introduction but should be given greater emphasis throughout the manuscript. The statement in the third paragraph of the introduction line 109 (“All together, these data suggest bats are playing an important role in the diffusion and persistence of rhabdoviruses…”) is crucial and should be moved earlier to frame the entire study. The evolutionary and viral emergence importance of bats as reservoirs for rhabdoviruses warrants further discussion to underscore the relevance of the tool being developed.*

Following the different comments above of Reviewer 2, we modified the updated version of the manuscript, including a specific paragraph in the Introduction section to emphasize the potential zoonotic risk associated with bat rhabdoviruses and to highlight the importance of having a broad-spectrum molecular detection technique for these viruses available: “All together, these data suggest that bats are playing an important role in the diffusion and persistence of rhabdovirus, with potential risks of human exposure. Apart from bat lyssaviruses, recent examples of potential zoonotic bat rhabdoviruses have been described. For examples, members of genus Ledantevirus identified in bats in China (with Rhinolophus rhabdovirus DPuer in Rhinolophus affinis) and Ghana (with Kumasi rhabdovirus in Eidolon helvum) have shown the ability to infect human cells in vitro (Binger et al., 2015; Li et al., 2022). In addition, populations living near these bats showed specific antibodies, demonstrating natural exposure to these viruses. The risk of transmission of bat rhabdovirus was also underlined with the identification of American bat vesiculovirus (ABV) in bats which were in contact with humans in the USA (Hause et al., 2020).”

*Lack of Discussion on Viral Evolution: While the manuscript notes the diversity of the Rhabdoviridae family, there is insufficient discussion on how this diversity contributes to viral evolution, including potential for zoonotic spillover or adaptation. A deeper exploration of how understanding rhabdovirus evolution could help mitigate risks associated with viral emergence would strengthen the manuscript.*

We also added some elements in the Introduction section, as follow: “Rhabdovirus evolution is characterized by frequent host changes and genetic diversification, due to the simplicity of their genomic structure and their high mutation rate (Walker et al., 2015). The discovery of new rhabdoviruses in wild animals, particularly in bats, raises concerns about their potential for transmission to humans and domestic animals. Their wide host range, ecological plasticity and ability to adapt to new environments highlight the importance of surveillance to identify any potential zoonotic emergence in the future.”.

*Line 479: “we did not have access to any cadavers or organs” but brains are mentioned in the results. Again, this is an interesting discussion point to include. What could it mean that all brains were negative, but virus was detected in the blood and saliva?*

According to the comment of Reviewer 2, we have added the following sentence in the Discussion section; “Only a few brains from individuals collected in Algeria have been tested, all negative, but their small number does not allow us to conclude that MBV is not neurotropic.”.

*What is the connection between this vesiculovirus and potential spillover to other species? Is there a zoonotic threat? Make the connection between this vesiculovirus and potential spillover clearer.*

As suggested by Reviewer 2, we have modified the initial related paragraph in the Discussion section as follow: “MBV appears to be able to infect different species of bats, unlike the other bat vesiculoviruses identified to date. So far, its ability to infect other mammals including humans, remains to be determined. However, the presence of this virus has been observed in oral swab, suggesting that it could be excreted and transmitted via this mode, in addition to its circulating in the blood. These two factors, combined with the hypothesis that this virus can also be transmitted by arthropods, may increase the possibility of transmission to other animal species.”.

As we indicated in the discussion, the evaluation of the zoonotic threat will require further investigation, in particular testing the capacity of MBV to infect different cell types of various origins, in particular human.

*I don’t think the results of this study reflect a “large” circulation of MBV; the title should be edited. Maybe "regional" circulation; the word "large" is a little misleading since it can also imply high prevalence. or "broad geographic" circulation?*

As Reviewer 2 rightly pointed out, we have modified the title by replacing “large” with Broad geographical”.

*444-449: Interesting there doesn’t seem to be host specificity. How does this compare to other vesiculoviruses?*

Most bat vesiculoviruses seem to be host-specific. However, other vesiculoviruses have a wide range of hosts. As shown in Figure 3, vesiculoviruses in phylogroups I and II appear to be arthropod-borne viruses, with some capable of infecting humans and livestock. We have added the following sentences in our revised manuscript: “This ability to infect different species of mammals is also found in most other arthropod-borne vesiculoviruses, infecting birds and reptiles and different species of mammals, especially livestock, in endemic areas.”.

*500-501: I don’t disagree that this is possible but generally bat flies are pretty host specific. This seems likely to happen occasionally but not regularly.*

We agree with Reviewer 2 and we have added the following sentences for this part: “…and may be in close contact. However, as most bat flies remain host-specific to their host, further research is needed to assess this hypothesis.”.

*Curious that none of them are positive for a lyssavirus given they were part of a screening program in Europe; were lyssavirus positive samples excluded?*

As previously mentioned, bat specimens that tested positive for rabies were excluded from our study, as we were looking for other rhabdoviruses. The brain samples from a limited number of positive individuals were only used for the validation method step.

Editorial and Data Presentation Modifications?

*Reviewer #1: Please revise the "saliva samples" to oral swab! In fact, when swabbing bats, not only will there be saliva in the sample, but also cells and potentially remnants from ingested food, in bats most likely insects.*

We thank Reviewer 1 for this precision. We modified the manuscript accordingly, replacing “saliva samples” with “saliva swabs” or “oral swabs”.

*L 69: Please reconsider the use of virus vs. virus species. The latter is only the taxonomical unit, but is not "isolated" or can cause disease.*

We modified the text of the manuscript accordingly.

*L 282/283: sensibility should be sensitivity*

Corrected.

*L 487: Remove In addtion*

Corrected.

*Reviewer #2: There’s a lot of repetition within the text across different sections, and the writing could overall be more concise. There are also minor grammatical errors throughout the text. Some are highlighted below.*

As we indicated previously for a similar comment by reviewer 2, we have lightened the text, removing as many repetitions as possible in the manuscript, and moving some information in the Supplementary Materials section.

*65-67: This is kind of vague and bland; can the authors connect it more directly to their study?*

We have included the following sentence in the Introduction section: “For example, the *Miniopterus schreibersii* bat species has an extensive geographical distribution, occurring in the Middle East, in Central to Southeast Asia, in North Africa and in southern Europe, with the largest populations found in the warmer Mediterranean area.”.

*79-80: Lyssaviruses are very extensively investigated in bats; I’d add the caveat Rhabdoviridae outside of lyssaviruses.*

Modified accordingly.

*Line 81-112: this can be more concise*

As suggested by reviewer 2, we have shortened this part.

*124: Should be “circulates” because the subject of the sentence is singular.*

Modified accordingly.

*152-154: subordinate clause is missing a verb.*

We added “including” before ”the frugivorous bat family…”.

*282-283: Test sensitivity instead of sensibility?*

Modified accordingly (see also reply to comments of Reviewer 1).

*313-315: Not sure what this means; what is interesting here?*

In this section, we wanted to emphasize the persistence of the infection over this two-year period. We have modified the text by adding at the end: “highlighting the persistence of the infection over the two-year period”. In addition, we have deleted the sentence ”All bat samples and other available and tested samples from these six caves were negative”.

*319: check the grammar*

We modified this sentence as follow: “. After a final verification step carried out by mapping using the completed sequence consensus as a reference, 16…”.

*323: typo*

Corrected (added missing parenthesis).

*356: define ABV*

Modified accordingly.

*400: higher than those found in other studies?*

Modified accordingly.

*404: Plecotus auritus is misspelled.*

Modified accordingly.

*487: A sentence is began “In addition” but never completed.*

Corrected.

Summary and General Comments

*Reviewer #1: The authors developed a novel nested realtime PCR, screened existing sample sets from bats from various countries and identified novel rhabdoviruses, surprisingly at a low prevalence. Using state-of--the-art technologies the authors further characterised the identified sequences as a novel virus.*

*I congratulate the authors to this very fine work. In all sections, i.e. introduction, materials and methods, results and discussions, the authors provide a great level of detail and scientific accuracy.*

We are very grateful to Reviewer 1 for the positive and constructive comments, as well as for the time spent evaluating and improving our manuscript.

*Reviewer #2: Note: This review was conducted by a PI and two PhD students (hence the inconsistent use of plural and singular first person). We met to discuss our reviews and comments from all three people are aggregated.*

*The manuscript investigates the development of a novel molecular detection tool designed to identify rhabdoviruses in animal populations, with a particular focus on bat species. Using a combined nested RT-qPCR technique, the authors aim to address the significant challenge of rhabdovirus diversity, which complicates the detection of these viruses. The study attempts to bridge a gap in molecular diagnostics for rhabdoviruses, which are important pathogens for public health and veterinary concern, particularly in the context of viral emergence and zoonotic transmission. This manuscript presents a novel diagnostic tool and straightforward results; this paper would be valuable to the broader scientific community.*

*1. Novelty and Innovation: The development of a broad-spectrum molecular tool for the detection of rhabdoviruses for use across animal species is a valuable contribution to the field, particularly given the high genetic diversity within the Rhabdoviridae family. The use of nested RT-qPCR represents a solid technical approach for addressing this challenge that could be applied in laboratory settings with minimal equipment requirements.*

*2. Relevance to Public Health: The manuscript addresses an important gap in diagnostics for rhabdoviruses, which pose significant threats to both human and animal health. The potential for zoonotic transmission is a critical concern, and the development of a reliable screening tool could significantly improve monitoring efforts.*

*In general, we are excited about the diagnostic tool. The authors did an admirable job of vetting it to confirm it is truly pan-rhabdovirus and show interesting results of a new vesiculovirus circulating in Mediterranean bats. We think it will make a valuable contribution to the literature but recommend greater clarity about the sample preservation and lack of discovery of lyssaviruses or rhabdoviruses in the brains. We also recommend the authors streamline the manuscript to focus on the applicability of their diagnostic test and its potential role in understanding potential viruses of concern for bat or human health. (Or not! I don't know whether vesiculoviruses are likely to be zoonotic.)*

We are very grateful to Reviewer 2 and to the two PhD students for their positive and constructive comments and for the time he devoted to evaluating our manuscript, which greatly improved it. We have taken these comments into account and have tried to respond and make corrections to our manuscript as much as possible and as appropriate.
